# A comparative analysis of factors influencing colorectal cancer’s age standardized mortality ratio among Korean women in the hot and cold spots

**DOI:** 10.1371/journal.pone.0273995

**Published:** 2022-09-09

**Authors:** ChuelWon Lee, SungMin Kim, JaeHyun Woo

**Affiliations:** 1 Medical Device Industry Program in Graduate School, Dongguk University-Seoul, Seoul, Korea; 2 Department of Medical Biotechnology, Dongguk University-Seoul, Seoul, Korea; Tabriz University of Medical Sciences, ISLAMIC REPUBLIC OF IRAN

## Abstract

The study aimed at exploring factors that most influence colorectal cancer (CRC) age standardized mortality ratio (ASMR) among Korean women, as reported in previous studies. The factors used the data of 250 municipalities from the Korean Statistical Information Service (KOSIS) from 2010 to 2018. In the exploratory survey, over 70% of women aged 65 and above died of colorectal cancer. After investigating the existing literature and theories, 250 regions were classified into hot and cold spots according to age standardized mortality ratio (ASMR). The Nearest Neighbor Index (NNI), Moran’s I index and The Durbin-Watson test were also utilized. The ASMR’s regional cluster analysis showed that the inland areas were the hot spots and the cold spots were in the southwest coastal areas. The result also showed the differences in dwellers’ lifestyles between these two regions as well as the mean difference between the two. In addition, there was no significant difference in ASMR for breast cancer, CRC deaths, and agricultural product shipments between the two regions. In the multiple regression model, CRC mortality, diabetes, and CRC age standardized incidence ratio (ASIR) were analyzed as major influencing factors, demonstrated a significant result with 30.6% by examining the adjusted R-squared. However, this study showed that factors such as smoking, alcohol consumption, abdominal obesity, breast cancer, and food consumption indicated to have less influence on the occurrence of CRC. The aging rate, amount of food consumption, seafood production, livestock product shipments, and drinking rate were higher in the cold spot than in the hot spot.

## Introduction

The world is challenged by the emergence of colorectal cancer (CRC) of which its cure has become the unceasing quest for both the sick and the doctors, medical device programmers, and other scientists who are trying to find solutions to the existing health concern. In 2018, the National Cancer Information Center (NCIC) in South Korea reported CRC as the third highest death-causing illness among men and women [1]. Although the rate of CRC as a root cause of death has been declining since mid-1980s in other parts of the globe like America, the case of Asian countries including South Korea remains comparably high along with the countries in Eastern Europe [2, 3]. In 2018, the National Cancer Information Center (NCIC) reported that the CRC was the highest cause of death and illness among men and women in South Korea.

CRC is triggered by food consumption and other aspects of lifestyle that add to the risk factors for mortality, like the intake of red and processed meat, and the high content of smoke, alcohol, and caffeine [4–8]. Despite the protective measures to avoid cancer-related mortality, such as physical activity, eating nutritious fruits and vegetables, particularly fiber, magnesium, garlic, fish, vitamins, calcium, dairy products, starch, and folic acid intake [9, 10] are causes of illness such as breast cancer and diabetes which are closely related to CRC [11–14].

Although the protective measures against cancer related mortality are highly recommended by health professionals, the number of CRC deaths continues to rise. This has fueled research in the field, like the work that studied the epidemiological characteristics of the incidence of colorectal cancer in Jeju-do [15]. The study reported that the region has the highest obesity rate, high-risk drinking rate, and smoking rate among 16 provinces nationwide, which contributed to the occurrence of CRC. Another study reported the speedy increase in CRC is attributed to the increasing cases of colon cancer [16]. The incidence of CRC deaths in the seven metropolitan cities, including Busan, was estimated by using a spatial multilevel model from 2003–2009 [17]. In addition, the geographical distribution of male and female colorectal cancer was analyzed in Seoul and Gyeonggi Province in 2009–2013 with the spatial statistics technique. The high incidence of female CRC was studied in the northern and eastern regions of Gyeonggi and in some parts of Northern Seoul [18].

The existing studies have revealed that the severity of mortality in female CRC in Korea is currently rising, and the degree of mortality varies according to age and regional circumstances. Therefore, this study examined the regional-hot/cold spot differences in factors that most influence the age standardized mortality ratio (ASMR) of female CRC in Korea.

## Materials and methods

The researchers reviewed the related literature and investigated the factors and major theories of CRC. Thereafter, an exploratory study was conducted to confirm the age distribution of female CRC deaths. For the data related to the dependent variable or explanatory variable, statistical data is used for 9 years from 2010 to 2018 on the Korean Statistical Information Service (KOSIS).

### Review process

From the data taken from KOSIS, the research is gazes on the quantity of food consumption (fish, meat, vegetables) as well as the alcohol and cigar consumption as a hypothetical causes of mortality in the targe hot spots like Jangan-gu, Gwonseon-gu, Paldal-gu, and Yeongtong-gu in Suwon-si, Sangdang-gu, Seowon-gu, and Cheongwon-gu in Cheongju-si, Gyeryong-si, Boeun-gun, Goesan-gun, and the cold spots like the Jeongeup-si, Sunchang-gun, Seongdong-gu, Ongjin-gun, Jangseong-gun, Wando-gun, Sacheon-si, Sancheong-gun, Hwasun-gun (KOSIS, 2010–2018).

However, the KOSIS did not provide any Eco-friendly certified agricultural products shipment and eco- certified livestock starting 2010–2013 but it does not provide any individual data of the districts within the city so the data is distributed to every district for example in the city of Cheongju there are 4 districts; Heungdeok-gu, Seowon-gu, Sangdang-gu, Cheongwon-gu. The KOSIS started to provide a complete data starting from the year 2014. In 2014 the area in Cheongwon-gun and the near areas becomes Cheongju city and the names of the area changes as well as the land area gets bigger comprising the city so there is a little difference between the data provided by the KOSIS when the place was still Cheongwon-gun and the recent data when it already become a city. This preprocess was edited by Microsoft Office 365 Excel program. The construction of the spatial data frame for the dependent variable is based on QGIS 3.8. This geographic information system (GIS) program is also used to visually verify the identification of errors in statistical analysis results. To confirm the concentration of the approximate age-standardized mortality ratio of female colorectal cancer, ASMRs from 250 cities and towns across South Korea are visualized by classifying them into 2 stages, respectively.

### Data analysis

The dependent variables are the mean of CRC’s ASMR for 9 years from 2010 to 2018 and independent/explanatory variable is year, city, gender, and cause of death. The spatial weight of the dependent variable is calculated based on the adjacency of the borders between regions. Moreover, the presence or absence of autocorrelation is determined by calculating Global Moran’s I value. The local cluster pattern analysis of the dependent variable is identified as the hot spot area and the cold spot area using Getis-Ord Gi*. For this calculation and identification, the statistical analysis programs R version 3.3.1 and RStudio version 1.1.463 were run on the Windows 10 Pro, 64-bit operating system platform.

**Fig 1:**
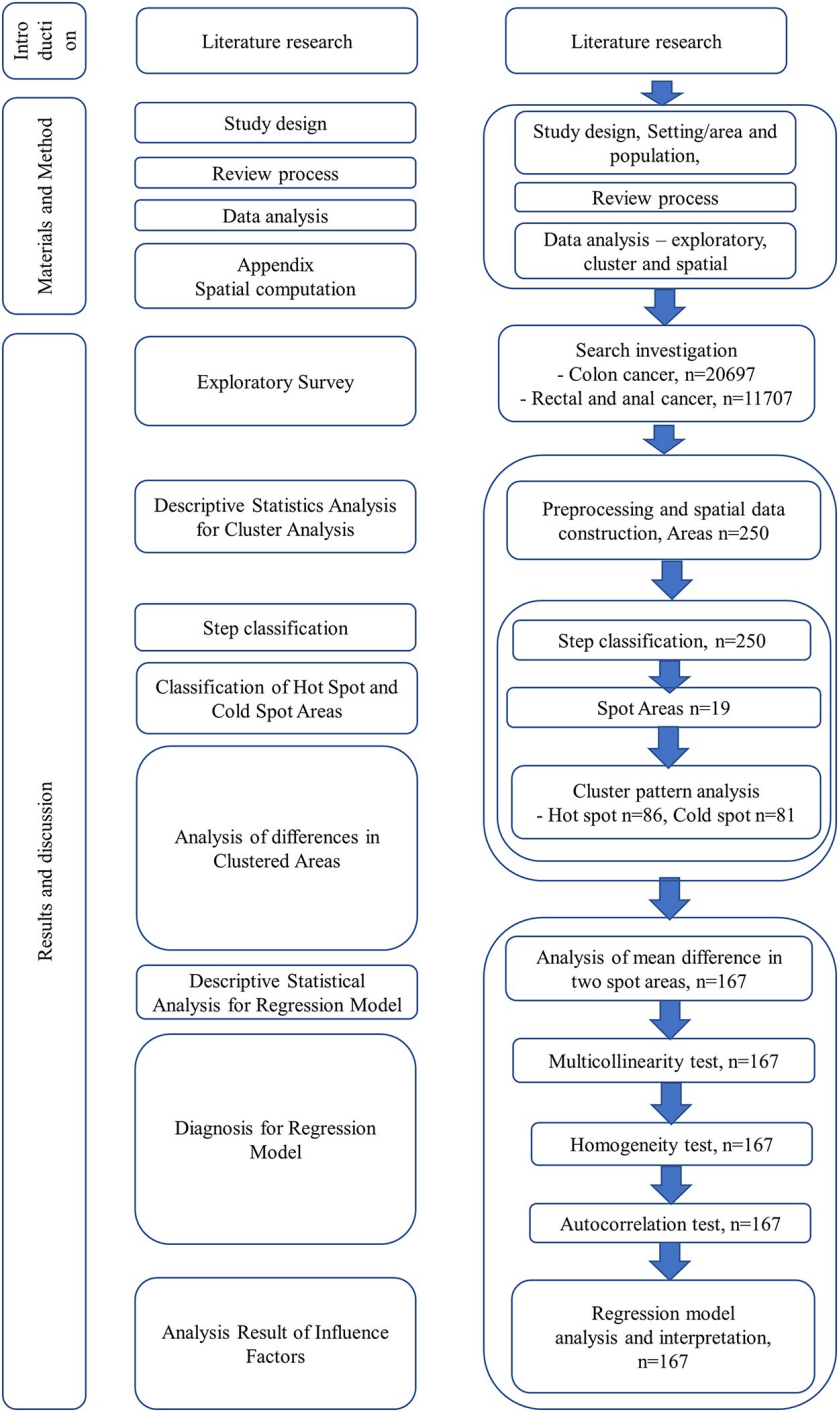
Flow chart for study method. It is the schematic diagram for this research method. After investigating the existing literature and theories, 250 regions were classified into hot and cold spots according to age standardized mortality ratio (ASMR), and the mean differences in causes were analyzed. The causes of these spots are also analyzed and interpreted.

To find the average difference between the two cluster regions, the mean of each explanatory variable, the equivariance by F-test, and the probability of significance by T-test were calculated. The regression model is used to support the statistical correlation of the CRC death factors. A test on explanatory variables is conducted to analyze this regression model along with the differences between the two regions. First, a multicollinearity test was conducted to examine the linear correlation. Second, the variance is tested in three ways to demonstrate the uniformity of data variance in two different cluster regions. Third, the Durbin-Watson test is performed to diagnose the autocorrelation of this model. After the explanatory variable test, the factors of this regression model are analyzed, and the results are interpreted. Fig 1 is a schematic diagram of the research method.

## Results

### Exploratory survey

The number of deaths by age 5 years gap is explored to determine the more precise age group of female colorectal cancer mortality [19]. As shown in Tables 1 and 2 finding, the elderly aged 65 years or older accounted for more than 70% of the total mortality; among the deaths of the colon, rectal and anal cancers.

**Table 1:** Summary statistics of mortality by colon cancer according to age groups.

2010–2018	Total mortality (min / max)	Mean ±SD	SE	Mortality (%)
Total	20698 (2094 / 2579)	2299.78 ±155.98	51.99	100.0
0~39 old	304 (0 / 30)	3.75 ± 6.68	0.74	1.47
40~64 old	4252 (30 / 162)	94.49 ±38.47	5.73	20.54
65~84 old	11662(139 / 553)	323.94 ±116.11	19.35	56.34
85 old or older	4479(94 / 450)	248.83 ±107.49	25.34	21.64

SD: standard deviation, SE: standard error, min/max: minimum/maximum

This table is a table analyzed by dividing the deaths of colon cancer (C18) into four age sections using S1A Dataset in S1 File.

**Table 2:** Summary statistics of mortality by rectal and anal cancers according to age groups.

2010–2018	Total mortality (min / max)	Mean ± SD	SE	Mortality (%)
Total	11707(1235 / 1350)	1300.78 ±37.17	12.39	100.0
0~39 old	190 (0 / 15)	2.35 ±4.25	0.47	1.62
40~64 old	2710 (19 / 97)	60.22 ±25.11	3.74	23.15
65~84 old	6441 (84 / 287)	178.92 ±60.11	10.02	55.02
85 old or older	2365(53 / 234)	131.39 ±59.41	14.0	20.20

SD: standard deviation, SE: standard error, min/max: minimum/maximum

This table is a table analyzed by dividing the fatalities of rectal and anal cancer (C19-C21) into four age sections using S1B Dataset in S1 File.

### Descriptive statistics analysis for cluster analysis

The observations in this study are organized by region into 250 municipalities across the country. Its mean is 8.15 and its standard deviation is 1.35.

#### Step classification

To visually examine the regional pattern of the age standardized mortality ratio in female CRC, 250 regions are divided into two steps. The ASMR of female colorectal cancer showed that the central region was higher than the southern region. In addition, to identify patterns with quantitative values, the distance between the 250 administrative districts of the county and the center point of the county districts was calculated by the NNI of Eqs (3)–(5).

The NNI of the regions was 1.15, the z value was 4.62, and the p value was <0.001. Since the probability of significance was significant at 95% CI, it was found that it had a random pattern.

### Diagnosis of spatial autocorrelation of dependent variables

The global Moran’s I value was calculated by using Eq (6) to confirm the presence of clustering of female CRC age standardized mortality ratio in municipalities across the country and to quantify spatial distribution patterns. The weight of the Moran’s I value was calculated by using the Queen method to determine whether the borders between regions are adjacent. Moran’s I by contiguity weight matrix shows that the Moran’s I value of the dependent variable by adjacency weight is 0.117 (SD: 1.77, p = 0.038, expectation: -0.0051, variance: 0.00472). Since this value is close to 0, spatial autocorrelation has a weak positive (+) random spatial pattern, and it is significant at the 5% significance level.

### Classification of hot spot and cold spot areas

As the ASMRs in the municipalities of the country have random spatial distributions, Getis-Ord Gi* was used in Eq (7) to identify hot spots and cold spot areas. The 250 regions were divided into 3 hot spots, 3 cold spots and 1 other area by z value. The breaks for z value were min, -2.58, -1.96, -1.65, Not significant, 1.65, 1.96, 2.58, and max. In addition, the clustered regions were classified into three categories; z = 1.64, z = 1.96 and z = 2.58.

Table 3 is a table that identifies areas with a z value of ±1.96 or higher as 10 hot spot areas and 9 cold spot areas. The hot spot area had many inland areas, and the cold spot area had many southwestern coastal areas or in the surrounding coastal areas.

**Table 3:** Hot/cold spot areas.

Spot	Z-value	Areas
Hot	2.58 <=	Jangan-gu, Gwonseon-gu, Paldal-gu, and Yeongtong-gu in Suwon-si, Sangdang-gu, Seowon-gu, and Cheongwon-gu in Cheongju-si
	1.96 to 2.58	Gyeryong-si, Boeun-gun, Goesan-gun
Cold	<= -2.58	Jeongeup-si, Sunchang-gun
	-2.58 to -1.96	Seongdong-gu, Ongjin-gun, Jangseong-gun, Wando-gun, Sacheon-si, Sancheong-gun, Hwasun-gun

This table shows hot and cold spot areas with a z value of ±1.96 or higher.

### Definition of dependent and explanatory variables

In order to compare risk factors in the clustered areas of Korea, dependent and explanatory variables were selected in areas with high and low female CRC cancer incidence. The dependent variables are the CRC ASMR of the two regions. And the explanatory variables are the old age rate [20], the number of elderly people [20], the number of deaths [21], the smoking population of 66 years old [22], the target region’s drinking rate [23], the abdominal obesity of 66 years old [22], the ASMR of female diabetes [21], the age standardized incidence ratio (ASIR) of colorectal cancer [24], the ASMR for breast cancer [21], the consumption index [25–31], agricultural product shipments [32], fishing production [33], and the shipment of livestock products [34]. Table 4 below describes the variables used for regional difference analysis and regression model analysis.

**Table 4:** Definitions of variables.

Variables	Explanation
CRC ASMR	Age standardized mortality ratio (ASMR) per 100,000 women in colorectal cancer (C18-C20) In order to compare the death level between groups with different demographic structures, the standard population uses the 2005 resident registration age group as the mortality rate after removing the effect of the age structure on the mortality rate $ASMR=\frac{\sum \left(A\times B\right)}{C}\times 100,000\;(1)$ A: mortality by age B: Population by age of the standard population C: standard population
Aging rate	Percentage of female population over 65 years old in mid-year population
Aged population	Number of female populations over 65 years old
CRC mortality	Number of female mortality populations in CRC
Smoker	Number of smokers surveyed in women aged 66 years from the lifestyle assessment results for medical examination
Drinking rate	Percentage of people who have drank at least once per month in the last year
Abdominal obesities	Number of abdominal obesities surveyed in women aged 66 years from the lifestyle assessment results for medical examination
ASMR for diabetes	ASMR per 100,000 women in diabetes disease (E10-E14)
CRC ASIR	ASIR per 100,000 women in colorectal disease (C18-C21)
ASMR for breast cancer	ASMR per 100,000 women in breast cancer (C50)
Consumption amount	Regional consumption amount per trillion won in agriculture, forestry and fisheries.
Agricultural product shipments	Ton of agricultural product shipments
Seafood production	Kg of seafood production
Livestock product shipments	Ton of livestock product shipments

This table is the variables and the explanation of S11 Dataset in S1 File.

### Definition of dependent variables for cluster analysis

The clustering of age-standardized mortality ratio [21] for female colorectal cancer in national administrative districts is conducted to identify the family history and genetic factors mentioned in the introduction. This ASMR for CRC is the age-standardized mortality ratio for CRC(colon, rectal and anal cancers) based on 100,000 people. It shows the dependent variables used for visualization and spatial autocorrelation analysis in this paper.

The agricultural products like vegetables that are full of magnesium, calcium, starch(glucose) and folic acid e.g. garlic as well as the protein filled livestock product like red meat and processed meat are important variables because those are the foods that influence in the occurrence of the CRC that is not only shown as the result of this study but also in the previous studies.

### Analysis of differences in clustered areas

Ten hotspot regions and nine cold spot regions in Table 3 are created and compared by year. Seowon-gu, Cheongju-si was established in July 2014, and there are no data from 2010 to 2013. The hotspot area used 86 frames except for 4 frames in Seowon-gu and Cheongju-si, and 81 frames in the cold spot area. Table 5 shows the technical statistics for the hot and cold spot areas.

**Table 5:** Hot/cold spot areas.

Spot area	Hot: n = 86			Cold: n = 81		
Variables	Sum	SD	Mean	Sum	SD	Mean
CRC ASMR	882.8	3.9	10.3	501.9	3.9	5.4
Aging rate	-	9.9	16.1	-	7.4	29.4
Aged population	918530	4634	10681	798698	5206.6	8914
CRC mortality	1039	6.89	12.1	607	5.4	6.0
Smoker	619	10.2	7.2	266	7.9	1.0
Drinking rate	2641	20.3	30.7	4607	5.7	57.0
Abdominal obesities	4912	44.1	57.1	2014	38.8	12.0
ASMR for diabetes	1015.4	6.01	11.8	688.5	4.7	7.2
CRC ASIR	2392.2	7.63	27.8	1998	4.2	25.0
ASMR for breast cancer	624.4	4.24	7.26	585.2	4.6	6.3
Consumption amount	4.38	0.80	0.51	11.67	0.13	101416
Agricultural product shipments	218339	5669	2539	244065	2641.6	2509
Seafood production	22031825	367067	256184	895796017	22826041	229810
Livestock product shipments	164019	2670.4	1907	330497	656.7	2345

This table is the statistics for the spot areas in S11 Dataset in S1 File, and the names of the areas are mentioned in Table 3.

Table 6 shows the results of the F-test to compare whether the two clustered regions satisfy homoscedasticity. In this table, Var. Equal gave true or false depending on whether the significant probability of variables was 0.05 or more as a result of homoscedasticity. If true, the Two-Sample T-test was executed, and if false, the Welch Two-Sample T-test was executed.

**Table 6:** Variance comparison of spot areas.

*Num df: 80, **Denom df: 85	Var. p-value	95%CI	Rate of Var. (Var. F)	Var. Equal
CRC ASMR	0.910	0.66, 1.59	1.025	TRUE
Aging rate	0.009	0.36, 0.86	0.557	FALSE
Aged population	0.290	0.82, 1.95	1.262	TRUE
CRC mortality	0.026	0.39, 0.94	0.609	FALSE
Smoker	0.019	0.38, 0.92	0.593	FALSE
Drinking rate	<0.001	0.05, 0.12	0.078	FALSE
Abdominal obesities	0.251	0.50, 1.20	0.775	TRUE
ASMR for diabetes	0.030	0.40, 0.95	0.616	FALSE
CRC ASIR	<0.001	0.19, 0.46	0.296	FALSE
ASMR for breast cancer	0.522	0.75, 1.78	1.151	TRUE
Consumption amount	<0.001	1.65, 3.93	2.544	FALSE
Agricultural product shipments	<0.001	0.14, 0.34	0.217	FALSE
Seafood production	<0.001	2507.16, 5981.61	3866.968	FALSE
Livestock product shipments	<0.001	3.18, 7.58	4.898	FALSE

This table is F-test results for comparing the means of hot spots and cold spots using S11 Dataset in S1 File.

* Num df is numerator degrees of freedom, where “numerator” is the mean square for the whole model.

**Denom df is denominator degrees of freedom, where “denominator” is the mean square for error.

The results of Table 6‘s homoscedasticity is analyzed by means of the independent sample T-test of Table 7 to determine whether the mean was different between the two regions.

**Table 7:** Significant differences of hot/cold spot areas.

Variables	*t	**df	p-value	95%CI	Estimated mean (Cold, Hot)	Result
CRC ASMR	-6.71	165	<0.001	-5.27, -2.87	6.20, 10.27	TRUE
Aging rate	9.24	156.93	<0.001	9.79, 15.11	28.57, 16.12	TRUE
Aged population	-1.08	165	0.28	-2324.12, 683.89	9860.47, 10680.58	FALSE
CRC mortality	-4.81	159.58	<0.001	-6.47,-2.70	7.49, 12.08	TRUE
Smoker	-2.79	158.82	0.006	-6.69, -1.14	3.28, 7.20	TRUE
Drinking rate	11.50	98.90	<0.001	21.65, 30.68	56.88, 30.71	TRUE
Abdominal obesities	-5.004	165	<0.001	-44.98, -19.53	24.86, 57.12	TRUE
ASMR for diabetes	-3.97	159.88	<0.001	-4.95, -1.66	8.50, 11.81	TRUE
CRC ASIR	-3.34	132.84	0.001	-5.01, -1.29	24.67, 27.82	TRUE
ASMR for breast cancer	-0.05	165	0.96	-1.38, 1.31	7.22, 7.26	FALSE
Consumption amount	5.59	133.05	<0.001	60156.79, 126064.19	144082.43, 50971.94	TRUE
Agricultural product shipments	0.70	121.83	0.486	-868.03, 1816.67	3013.15, 2538.83	FALSE
Seafood production	4.26	80.04	<0.001	5755197, 15850855	11059210, 256184	TRUE
Livestock product shipments	3.03	109.9	0.003	752.00, 3594.02	4080.21, 1907.20	TRUE

This table shows the results of estimating the significant mean differences between hot and cold spots using S11 Dataset in S1 File.

* tmeasures the size of the difference relative to the variation in sample data.

**df is degrees of freedom which are the amount of information the data and this value is determined by the number of observations.

As shown in the difference between the two regions in Table 7, the two regions had significant mean differences in CRC ASMR, aging rate, CRC mortality, smokers of 66 years old, drinking rate, abdominal obesities of 66 years old, ASMR for diabetes, CRC ASIR, intermediate consumption amount, seafood production, and livestock product shipments. However, there are no significant differences in the aged population, ASMR for breast cancer, or agricultural product shipments in the two regions. Among the variables with significant differences, CRC ASMR, CRC mortality, smokers of 66 years old, abdominal obesities of 66 years old, ASMR for diabetes, and CRC ASIR had higher averages in the hotspot area. On the other hand, the aging rate, drinking rates, intermediate consumption amounts, seafood production, and livestock product shipments were higher in the cold spot area.

### Descriptive statistical analysis for regression model

The hot and cold spot areas applied in this study represent the summary statistics of the regression model in Table 8. The number of observations in this model is 167, and the definition of each of the dependent and explanatory variables is as mentioned in Table 8.

**Table 8:** Summary statistics of regression model.

n = 167	SD	Mean	VIF	1/VIF
CRC ASMR	4.41	8.32	-	-
Aging rate	10.77	22.20	4.30	0.23
Aged population	4922.05	10283	5.75	0.17
CRC mortality	6.62	9.843	2.27	0.44
Smoker	9.3	5.229	1.45	0.69
Drinking rate	19.95	43.4	2.03	0.49
Abdominal obesities	44.54	41.47	6.55	0.15
ASMR for diabetes	5.65	10.25	1.41	0.71
CRC ASIR	6.38	26.29	1.15	0.87
ASMR for breast cancer	4.39	7.24	1.15	0.87
Consumption Amount	115710.3	96133.3	2.84	0.35
Agricultural product shipments	4457.97	2769	1.55	0.65
Seafood production	16747916	5495975	1.72	0.58
Livestock product shipments	4655.22	2961	2.31	0.43

This table shows the statistical values of regression model analysis using S11 Dataset in S1 File. Also, this table shows the Variance Inflation Factor (VIF) and1/VIF in S11 Dataset in S1 File.

### Diagnosis for regression model

#### Multicollinearity test

Table 8 shows the results of this test to examine the linear correlation of explanatory variables. Since the largest value of the variance inflation factor (VIF) is 6.55 and all tolerance values are greater than 0.1, it was determined that multicollinearity is not a problem.

#### Homogeneity test

For the diagnosis of homoscedasticity using the null hypothesis, three tests of Kruskal-Wallis rank sum, Wilcoxon rank sum, and Fligner-Killeenare conducted, and the results are shown in Table 9. As a result of the tests, homoscedasticity exists because the P-value is greater than 0.05 as 0.1745, 0.1745 and 0.1834, respectively.

**Table 9:** Homogeneity test.

Test	Chi.	DF	P-value
Kruskal-Wallis rank sum	1.8438	1	0.1745
Wilcoxon rank sum	568808	-	0.1745
Fligner-Killeen	1.7697	1	0.1834

This table results the homoscedasticity for S11 Dataset in S1 File.

#### Autocorrelation test

The Durbin-Watson test confirmed whether the residuals were autocorrelated. As the results of this test, autocorrelation is -0.0328, DW statistic is 2.050599, and p-value is 0.766, those are not significant. Since the DW statistic is close to 2, there is no autocorrelation.

### Analysis result of influence factors

The analysis results of the regression model are shown in Table 10, and have 30.6% explanatory power by the modified decision coefficient (Adj_R^2^). In addition, since the p-value is less than 0.05 and the F-statistic is 6.619, it shows that this model is significant. The statistics of the residuals show the normal distribution of the residuals, with a median of -0.211, slightly skewed to the left. Also, the size of 1Q is slightly larger than 3Q. Nevertheless, the distribution is symmetrical and is not greatly skewed to one side. Women with colorectal cancer ASMR in hotspot and cold spot regions have the number of deaths in female colorectal cancer (99.9% CI), diabetes (95% CI), female colorectal cancer ASIR (95% CI), and the aged population (99.9% Cl) was significantly affected.

**Table 10:** Result of regression model.

	Coefficients			
Variables	Estimate	Standardized	Std. Err.	P value
Aging rate	-9.767e-02	-2.383e-01	5.498e-02	0.078 (.)
Aged population	-5.056e-04	-5.650e-01	1.388e-04	<0.001(***)
CRC mortality	3.282e-01	4.917e-01	6.504e-02	<0.001(***)
Smoker	5.370e-02	1.136e-01	3.676e-02	0.15
Drinking rate	-1.615e-02	-7.317e-02	2.033e-02	0.43
Abdominal obesities	0.010	1.037e-01	1.637e-02	0.53
ASMR for diabetes	0.132	1.700e-01	5.984e-02	0.028 (*)
CRC ASIR	9.680e-02	1.400e-01	4.798e-02	0.045 (*)
ASMR for breast cancer	-3.177e-02	-3.157e-02	6.980e-02	0.65
Consumption amount	4.387e-06	1.152e-01	4.152e-06	0.29
Agricultural product shipments	-6.756e-05	-6.838e-02	7.955e-05	0.40
Seafood production	-1.167e-08	-4.437e-02	<0.001	0.60
Livestock product shipments	1.016e-04	1.074e-01	<0.001	0.28
Intercept	8.274	0.000	2.169	<0.001(***)
Model statistics	-	-	Residuals	-
R²	0.36	-	Min	-13.456
Adj_R²	0.306	-	1Q	-2.229
P-value	<0.001	-	Median	-0.211
F-statistic	6.619 on 13 and 153 DF	-	3Q	1.712

Note)

***, *,.; P<0.001, P<0.05, P<0.1 at significance level, This table is the result of regression analysis of S11 Dataset in S1 File.

The regression equation for this model is equal to Eq (2). This leads to predication that as these number of CRC mortality, ASMR for diabetes, and CRC ASIR increases, the ASMR for female colorectal cancer will increase. In addition, as the aged population increased, the standardized mortality rate for colorectal cancer decreased. This suggests that while the aged population increases, the ASMR of colorectal cancer will further decrease on a 100,000 basis.


$$y=\left(4.917e^{-1}\times CRC\;mortality\right)+\left(1.700e^{-1}\times ASMR\;for\;diabetes\right)+\left(1.400e^{-1}\times CRC\;ASIR\right)+\left(-5.650e^{-1}\times Aged\;population\right)$$ (2)

## Discussion

In previous studies, women’s age, abdominal obesity, meat, fish, and vegetable food, breast cancer, and diabetes were factors influencing CRC. According to some studies, there is rarely a link between CRC and alcohol consumption. This study shows that breast cancer and vegetable food had no effect on CRC ASMR. In addition, smoking and drinking, abdominal obesity, breast cancer, meat, fish, and vegetable food hardly affected CRC ASMR in Table 10. Therefore, diabetes was an important factor influencing CRC ASMR, which was commonly found in previous studies.

Also, this study showed the relatively significant differences between the two regions, such as aging rate, CRC mortality, smokers, drinking rate, abdominal obesity, diabetes, CRC ASIR, consumption amount, seafood production, and livestock products but it is the aged population, CRC mortality, diabetes, and CRC ASIR have the most important influence.

This study managed the differences between study setting and result. First, there was a change in administrative area and the estimation of some data as a limitation of data construction. This study analyzed presumptively the areas of Cheongju and Suwon among the administrative districts in the hotspot area on a nine-year basis. Second, the data on food consumed by the entire resident were compared because the exact data consumed by the population aged 65 or older in the hot spot and cold spot areas were not known.

## Conclusion

Diabetes is a common factor of CRC in existing studies and it is proven in this research. This research can be utilized as a foundation for diabetes prevention. In addition, the difference between the hot spot and the cold spot could be used as a mediation factor for the cluster in the future. Due to the limitations of the sizes provided by KOSIS, the SD values of smoker, agricultural product shipments, seafood production, and livestock product shipments are pretty small compared to the means. The findings of this study on the relation between the SD and the mean support the common trend of the mean increasing as the sampling distribution decreases.

The study found that diabetes was the most common factor in CRC. These two methods refer to regression analysis of available factors and the comparison of z values in the hot spot and cold spot areas. The age standardized mortality ratio in southwestern coastal areas or in the surrounding coastal areas is lower than the other areas in South Korea.

## Appendix

### Spatial computation

#### Nearest neighbor index

The Nearest Neighbor Index (NNI) analysis is a method that reveals whether the formation of spatial distribution patterns of points is functioning by interaction or by random formation. It can be seen from the R index of Eq (3) that the distance for each point of the point distribution pattern differs from the random point distribution pattern interval [35]. As in Eq (3), d_o_ is the average adjacent distance of the closest points to the observed points, and d_E_ is the average adjacent distance of the closest points to any points. It is the average adjacent distance between the closest points and arbitrary points. As in Eq (4), d_E_ can be obtained by the density of points by region. The z of Eq (5) is the normal standard deviation and is calculated by dividing the difference between the mean adjacent distances of d_o_ and d_E_ by the standard error (SEd_o_) of the average nearest distance. As a result, if NNI is 0, it becomes a clustered pattern, 1 is a random pattern, and 2.15 is a regular pattern. Also, in the 95% confidence interval (CI), a z value lower than -1.96 or higher than +1.96 indicates that the distribution was not randomly distributed [36].

The nearest-neighbour index is:

$$R=d_{O}/d_{E}$$ (3)

Where R is the index, d_o_ is the observed mean nearest neighbor distance, and d_E_ is the expected mean nearest neighbor distance for a random disposition of points:

$$d_{E}=1/2\sqrt{p}$$ (4)

Where p is the number of points divided by area, a test of significance is provided by:

$$z=(d_{O}-d_{E})/SEd_{O}$$ (5)

Where z denotes the normal standard deviation (the sampling distribution is normal) and SEd_o_ denotes the standard error of the mean nearest-neighbour distance [35].

#### The Moran’s I index

The existence of global autocorrelation can be quantified and generalized by the distribution pattern of individuals in the region through Moran’s I index [37]. The Moran’s I index is equal to Eq (6).


$$Moran^{'}s\;I=\frac{1}{S^{2}}\frac{\sum_{i=1}^{n}\sum_{j=1}^{n}W_{ij}(Z_{i}-\overset{-}{Z})(Z_{j}-\overset{-}{Z})}{\sum_{i=1}^{n}\sum_{j=1}^{n}W_{ij}}$$ (6)

n: number of observations

w_ij_: spatial weight between i and j area

In Eq (6), w_ij_ which is a component of the spatial weight value w, is assigned a weight value based on the whether the unit regions i and j share a boundary. It is 0 if not adjacent to each other and 1 if neighboring. The value of spatial region i is x_i_, and $\bar{x}$ is the average value of variable x. And n is the number of unit areas. The Moran’s I index represents a spatial cluster of heterogeneous attributes as it approaches -1, and a cluster of similar attributes as it approaches 1. Also, if the global Moran index is 0, there is no spatial autocorrelation.

The characteristics of Seoul’s building supply and changes in spatial clustering patterns were studied. Methods for analyzing the distribution pattern of spatial phenomena are global and local cluster pattern analysis. Global cluster analysis uses Getis-Ord General G(General G) or Moran’s I, and local cluster analysis uses the LISA index or Getis-Ord Gi*(Gi*). And the hot spot analysis uses General G와 Gi* [38].

Gi* calculates the z-value and p-value of the spatial unit in the target area as in Eq (7). Through this, it is possible to grasp statistically significant clustering tendencies of high attribute values (hot spots) and clustering tendencies of low attribute values (cold spots). It also has the advantage of being able to plot the analysis in spatial units [38].


$$G_{i}^{*}(d)=\frac{\sum_{j=1}^{n}W_{ij}X_{j}-\overset{-}{X}\sum_{j=1}^{n}W_{ij}}{WSD\sqrt{\frac{\left[n\sum_{j=1}^{n}W_{ij}^{2}-{(\sum_{j=1}^{n}W_{ij}^{2})}^{2}\right]}{n-1}}}$$ (7)

i, j: unit of analysis

x_i_, x_j_: attribute data of i and j area

w_ij_: spatial weight between i and j area

n: number of unit of analysis

WSD: weighted standard distance

### The Durbin-Watson test

The Durbin-Watson test is a test that is used to determine if the residual error satisfies the assumption of independence and whether autocorrelation exists. As in Eq (8), the statistic of this test is called d, and *e*_*t*_ is the residuals remaining after prediction. When the value of d is close to 0 or close to 4, autocorrelation exists between the residuals. Independence should be maintained between the residuals, and when there is no significant relationship between the residuals, it should come close to 2 [39].

$$d=\frac{\sum_{t=2}^{T}{\left(e_{t}-e_{t-1}\right)}^{2}}{\sum_{t=1}^{T}e_{t}^{2}}$$ (8)

*e*_*t*_ is the residual. Where *T* is the number of observations.

## Supporting information

S1 File Supplementary for the dataset in the manuscript. (DOCX)
