# Supplementary material for: A comparative analysis of factors influencing colorectal cancer’s age standardized mortality ratio among Korean women in the hot and cold spots
Source: PLoS One. 2022 Sep 9;17(9):e0273995. doi: 10.1371/journal.pone.0273995 (PMC9462820; doi:10.1371/journal.pone.0273995)

Content

[S1 Dataset 2](#_Toc112401259)

[S1A Dataset 2](#_Toc112401260)

[S1B Dataset 2](#_Toc112401261)

[S2 Dataset 3](#_Toc112401262)

[S2A Dataset 3](#_Toc112401263)

[S2B Dataset 3](#_Toc112401264)

[S2C Dataset 3](#_Toc112401265)

[S2D Dataset 3](#_Toc112401266)

[S3 Dataset 5](#_Toc112401267)

[S4 Dataset 7](#_Toc112401268)

[S5 Dataset 8](#_Toc112401269)

[S6 Dataset 9](#_Toc112401270)

[S7 Dataset 10](#_Toc112401271)

[S7A Dataset 10](#_Toc112401272)

[S7B Dataset 10](#_Toc112401273)

[S7C Dataset 10](#_Toc112401274)

[S7D Dataset 10](#_Toc112401275)

[S7E Dataset 11](#_Toc112401276)

[S7F Dataset 11](#_Toc112401277)

[S7G Dataset 11](#_Toc112401278)

[S8 Dataset 12](#_Toc112401279)

[S9 Dataset 13](#_Toc112401280)

[S10 Dataset 14](#_Toc112401281)

[S11 Dataset 16](#_Toc112401282)

# S1 Dataset

a. Original refence: [12] Death number and mortality rate by 236 death causes, gender and age of 5

## **S1A Dataset**

b-1. This dataset shows female fatalities and mortality rate by Colon Cancer(C18).


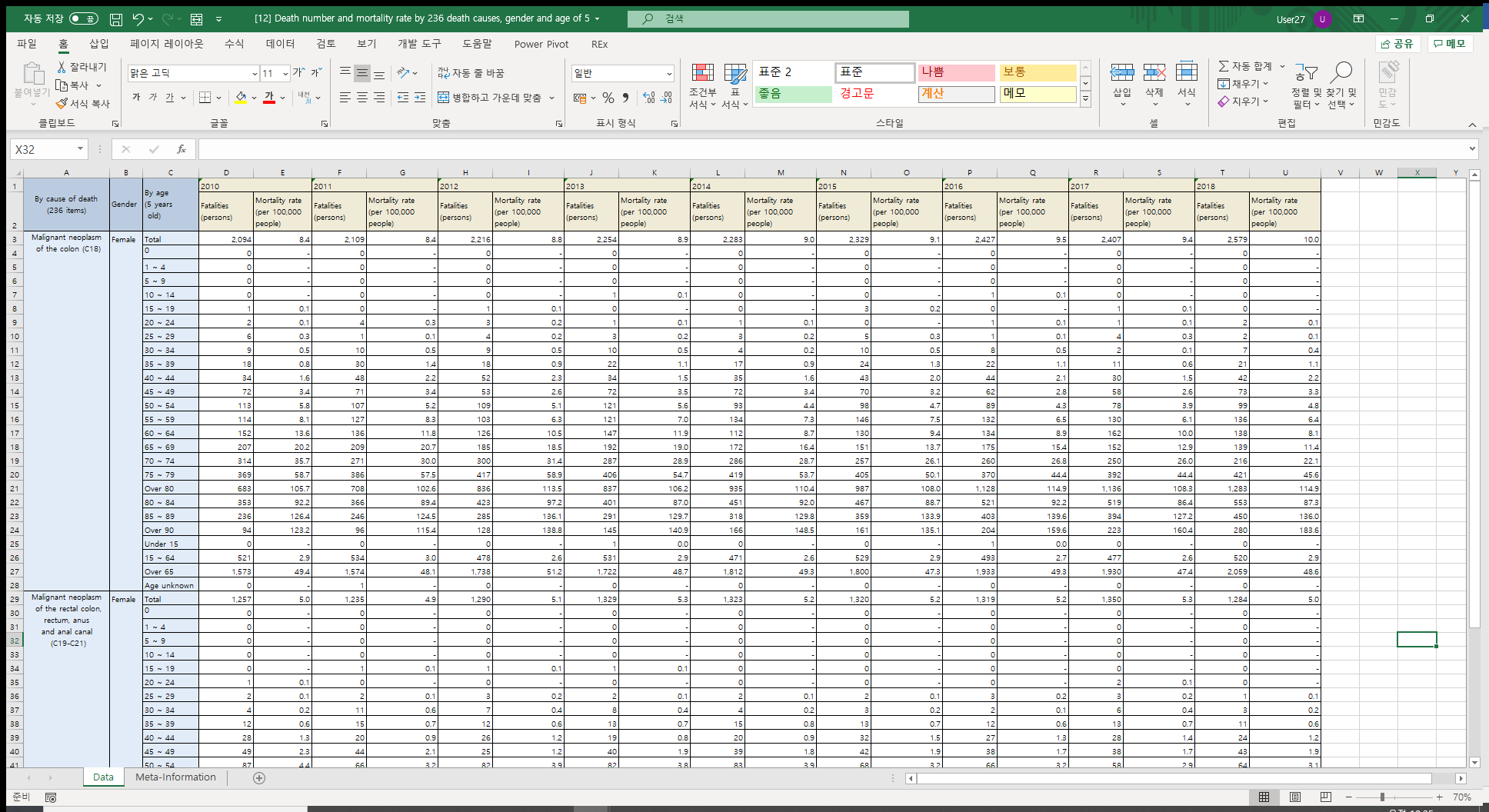


## S1B Dataset

c-1. This dataset shows female fatalities and mortality rate by Rectal and Anal Cancers (C19-C21).


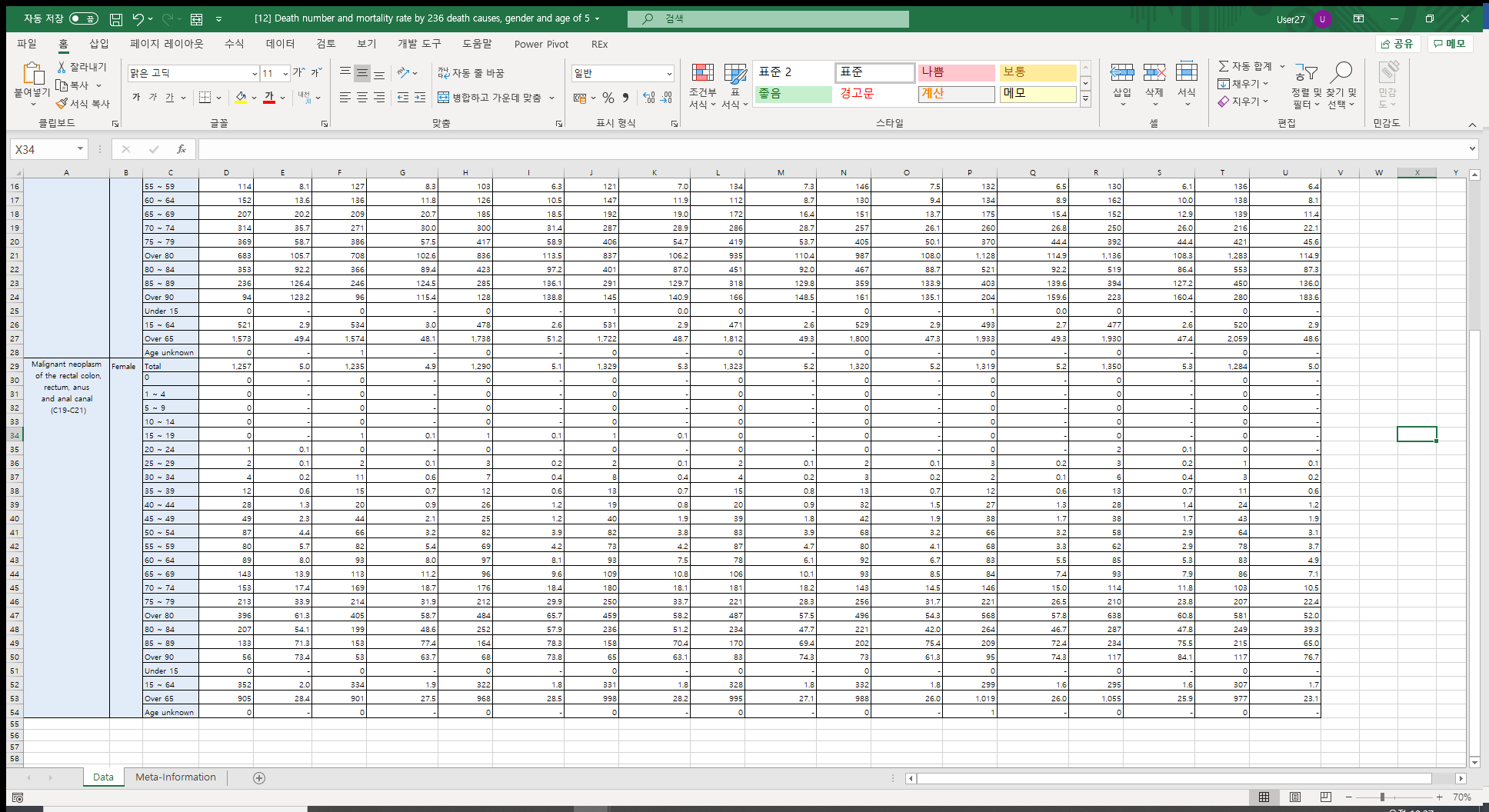


# S2 Dataset

a. original reference: [13] Death number, mortality rate and age standardized mortality ratio by 50 death causes

## **S2A Dataset**

a. sub-reference No.: [13-1]


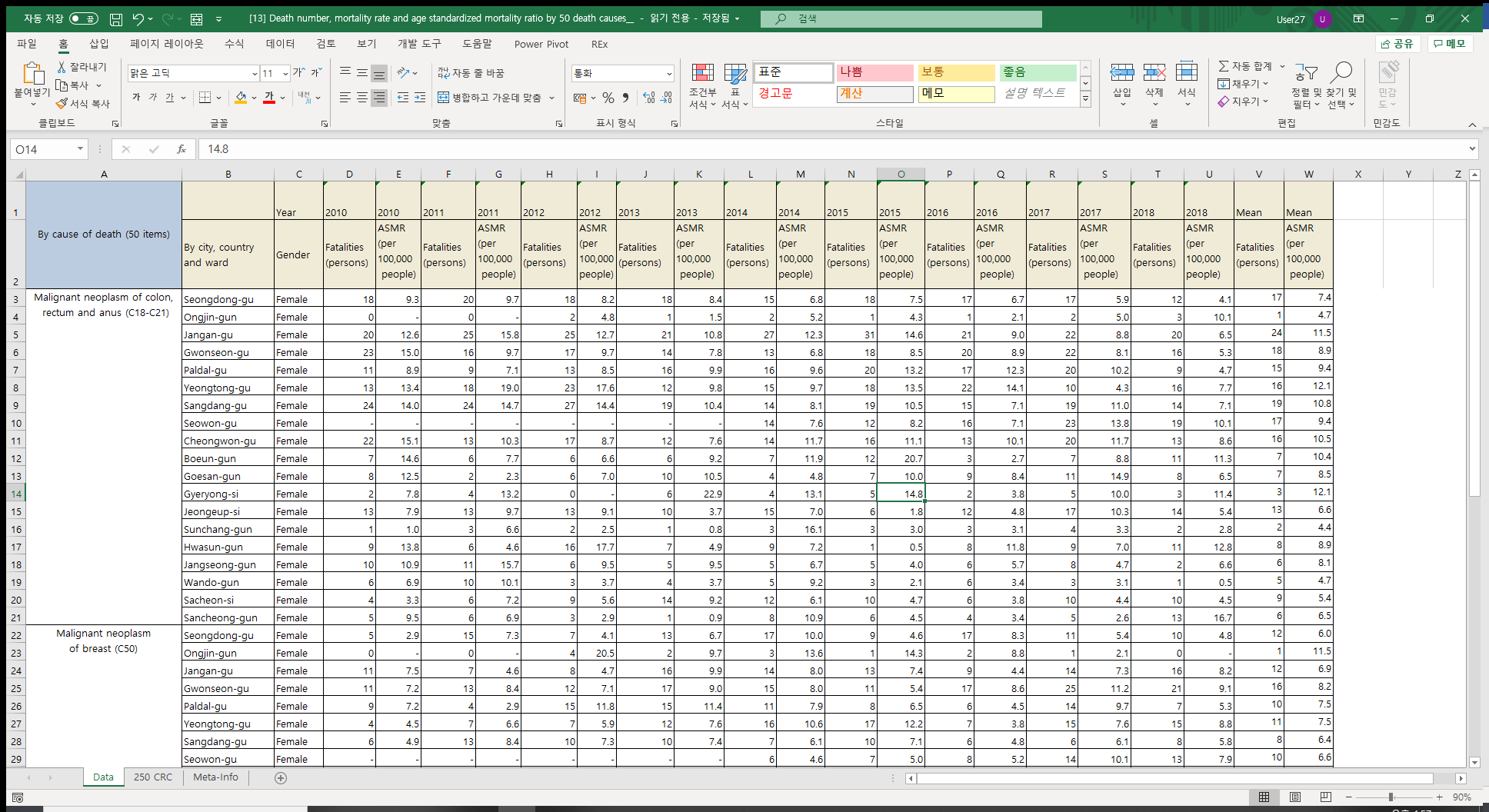


## S2B Dataset

a. sub-reference No.: [13-2]


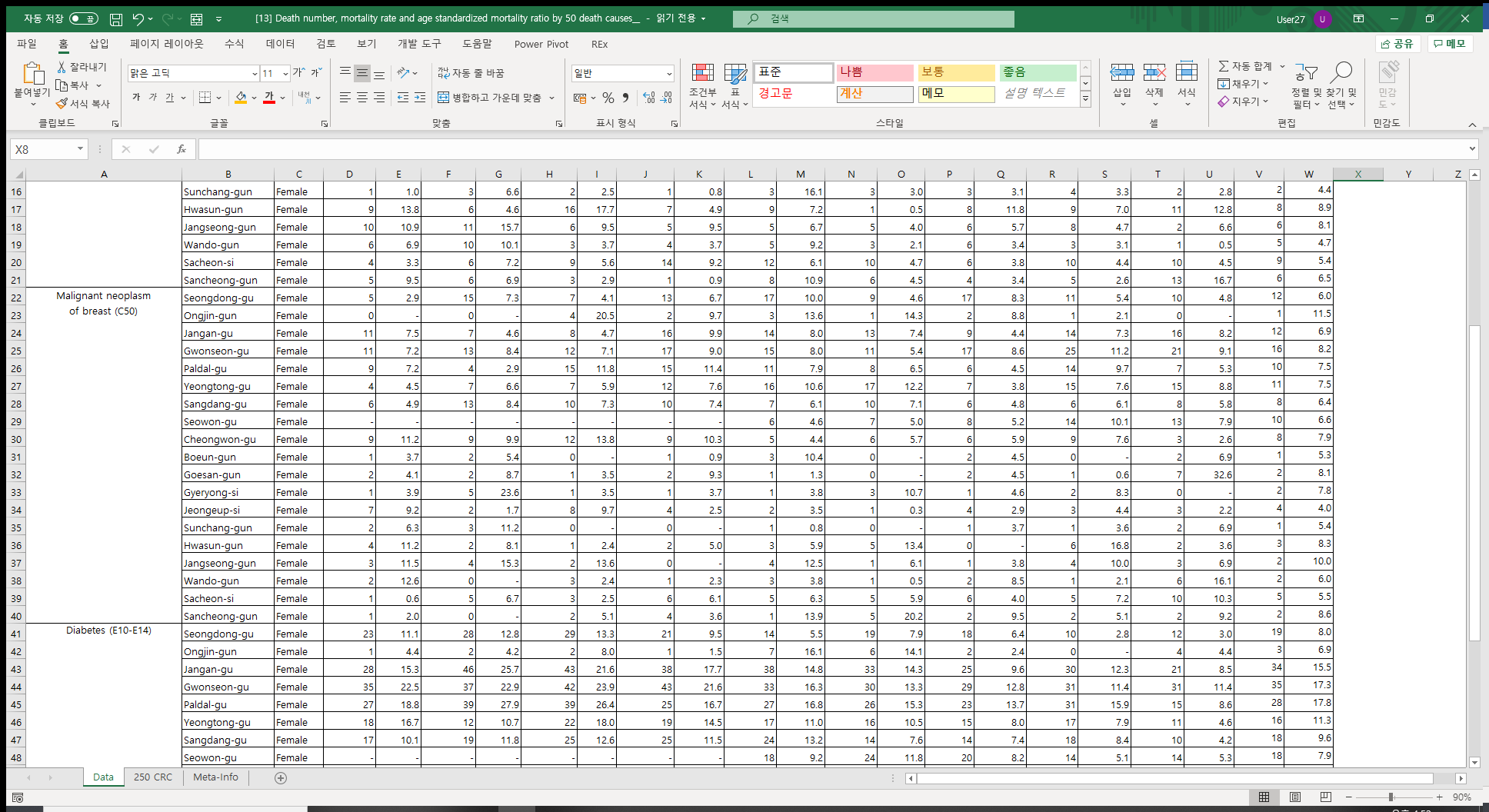


## S2C Dataset

a. sub-reference No.: [13-3]


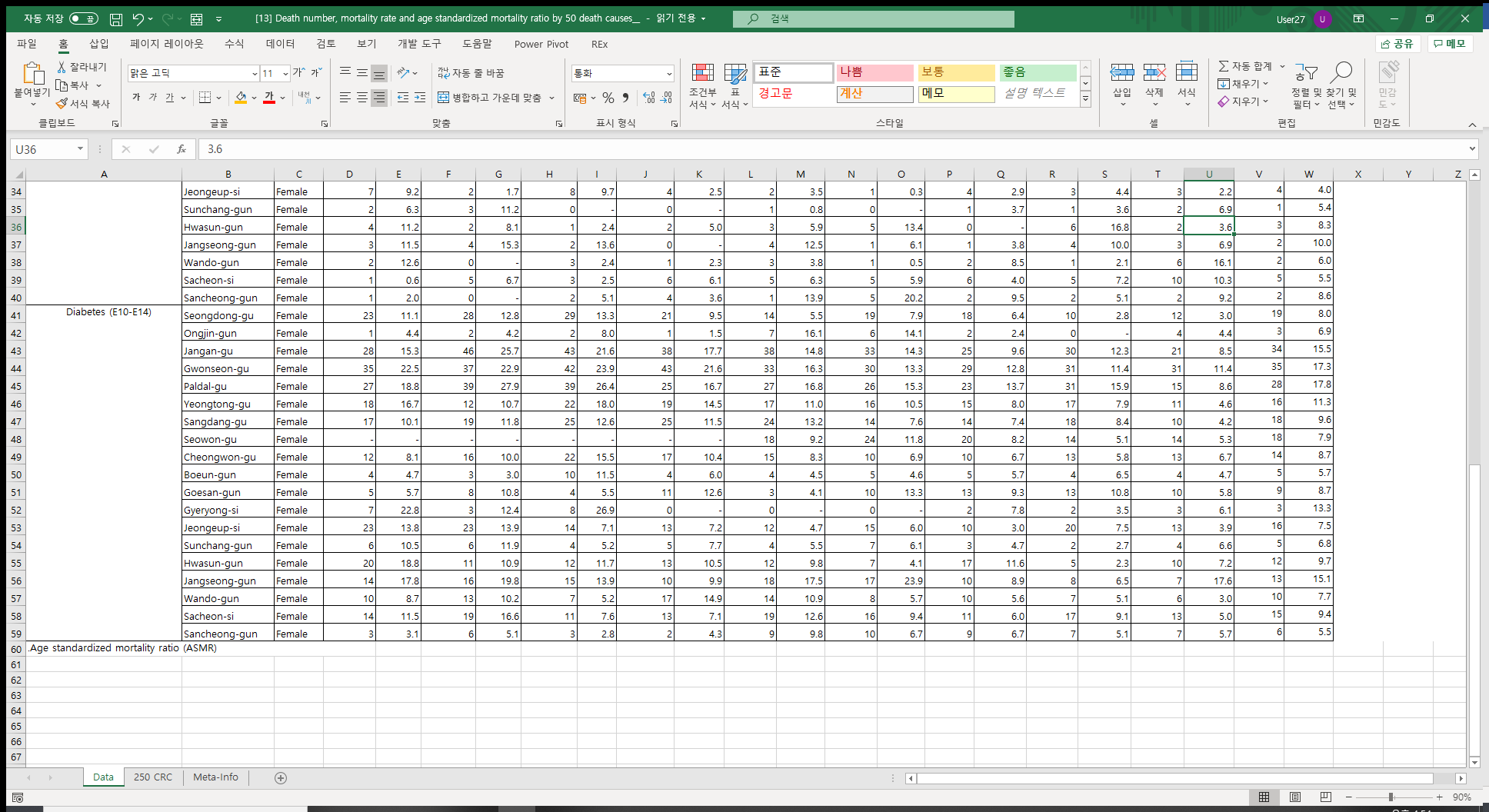


## S2D Dataset

a. sub-reference No.: [13-4]


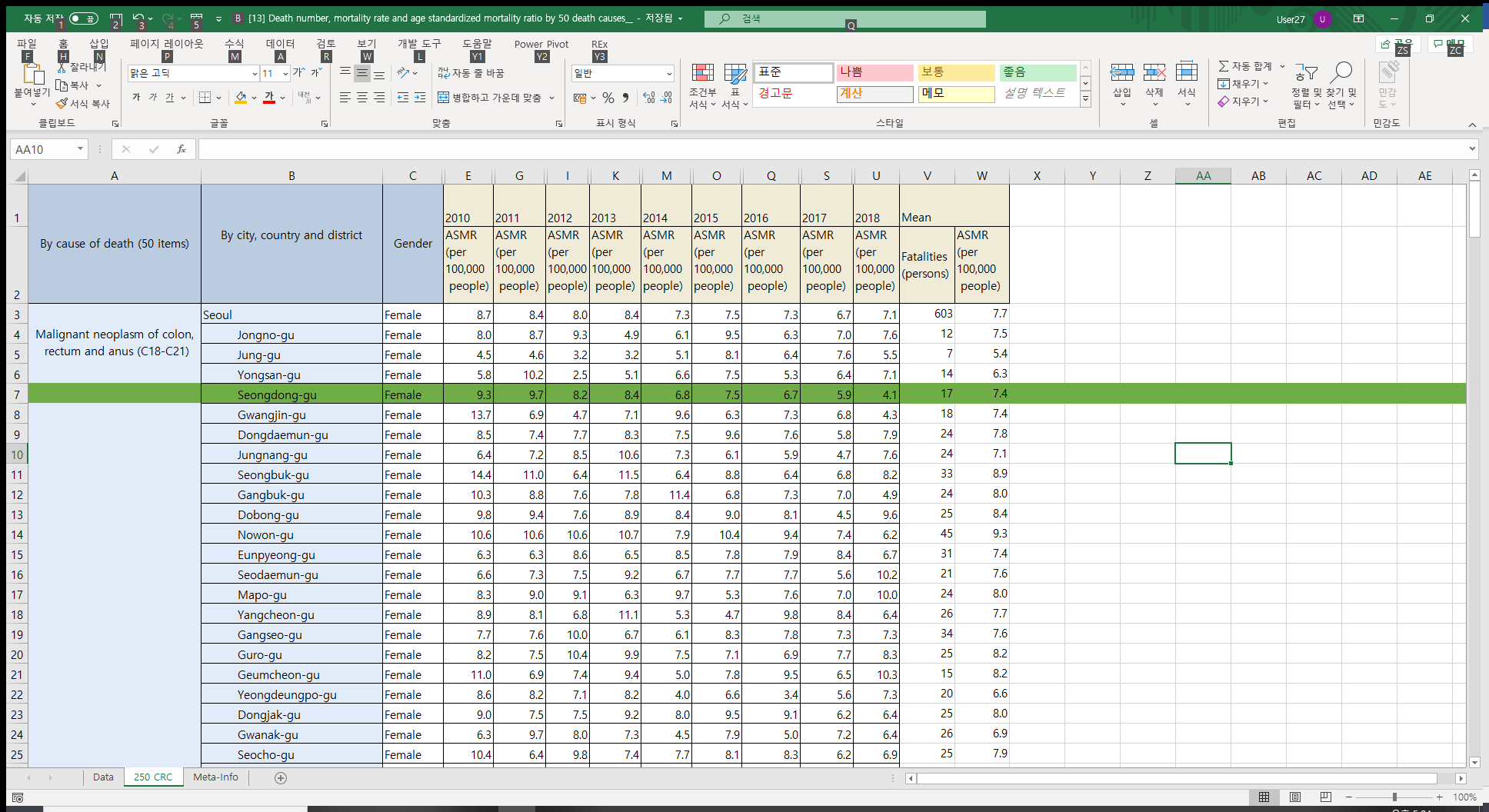

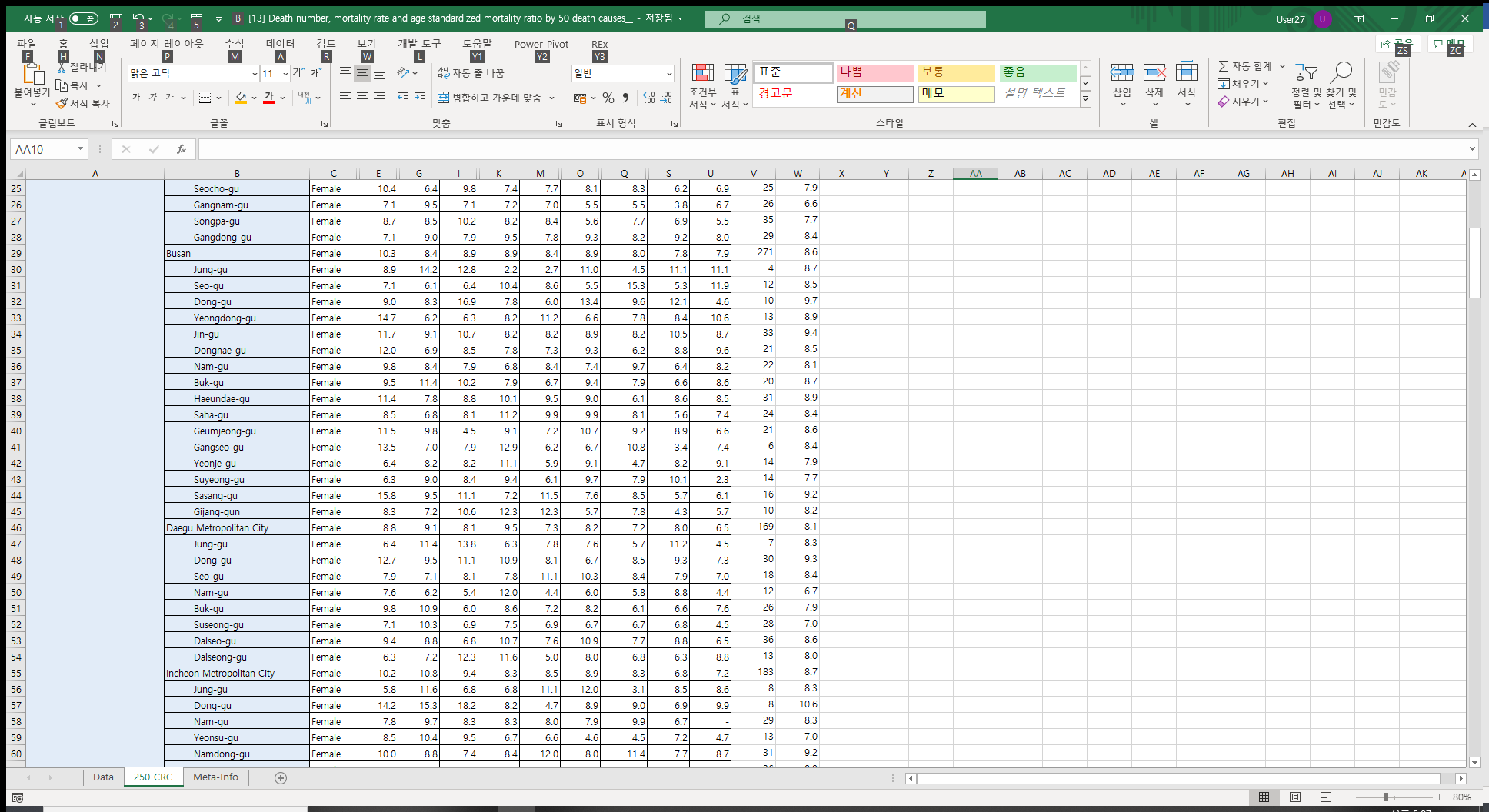


# S3 Dataset

a. original refence: [14] Resident registration population of the mid-year by city, province and age of 5


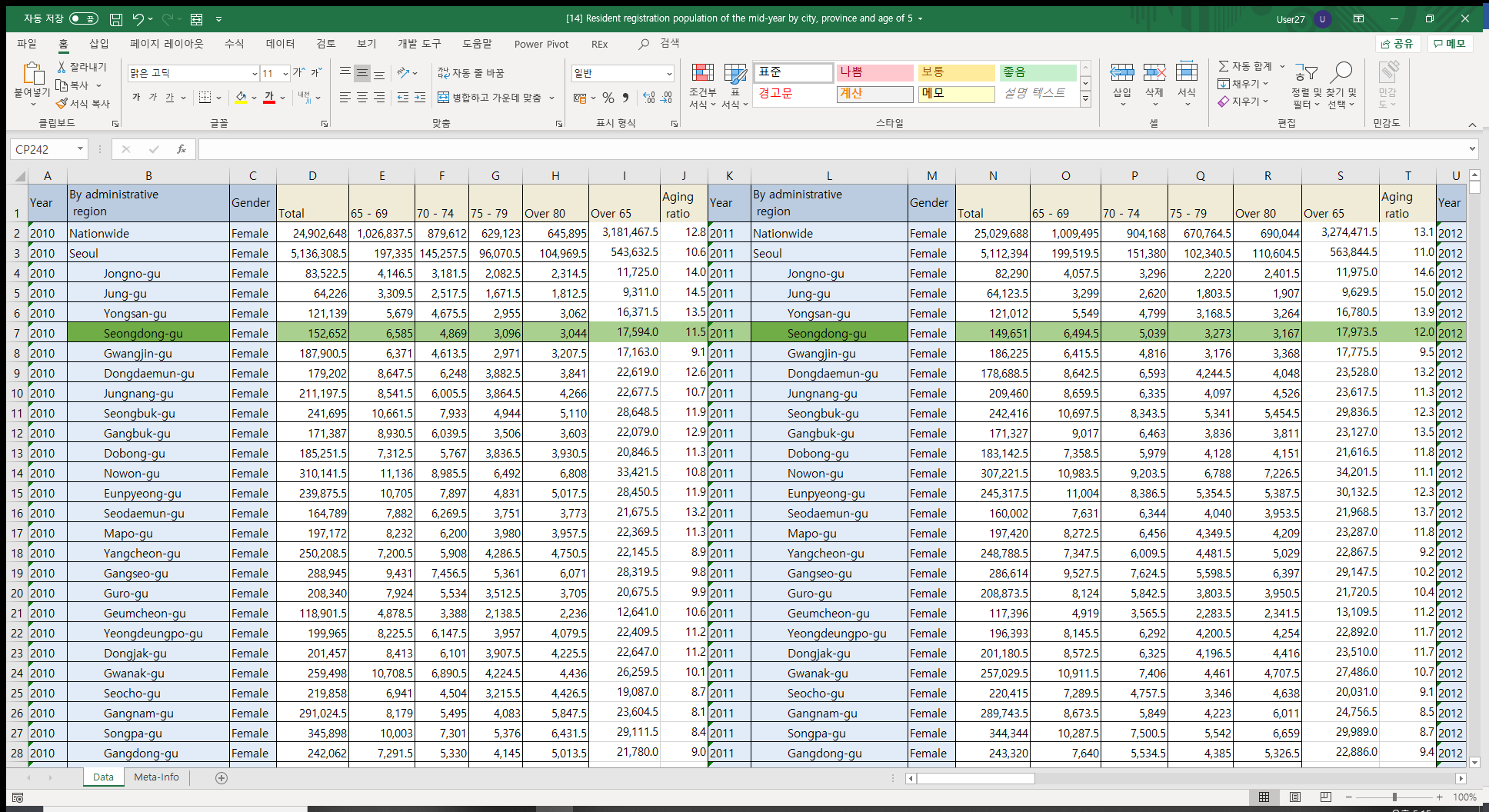


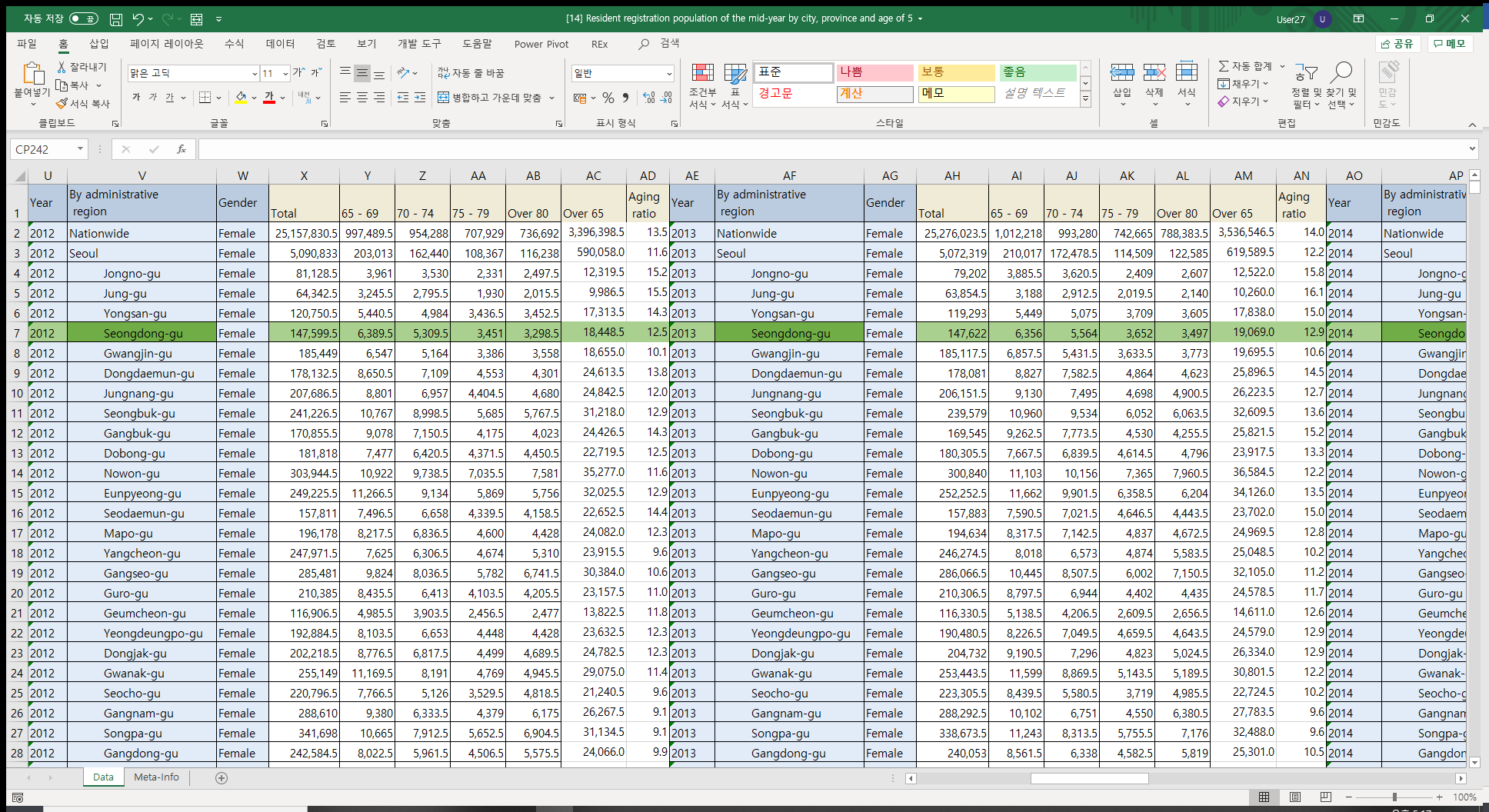


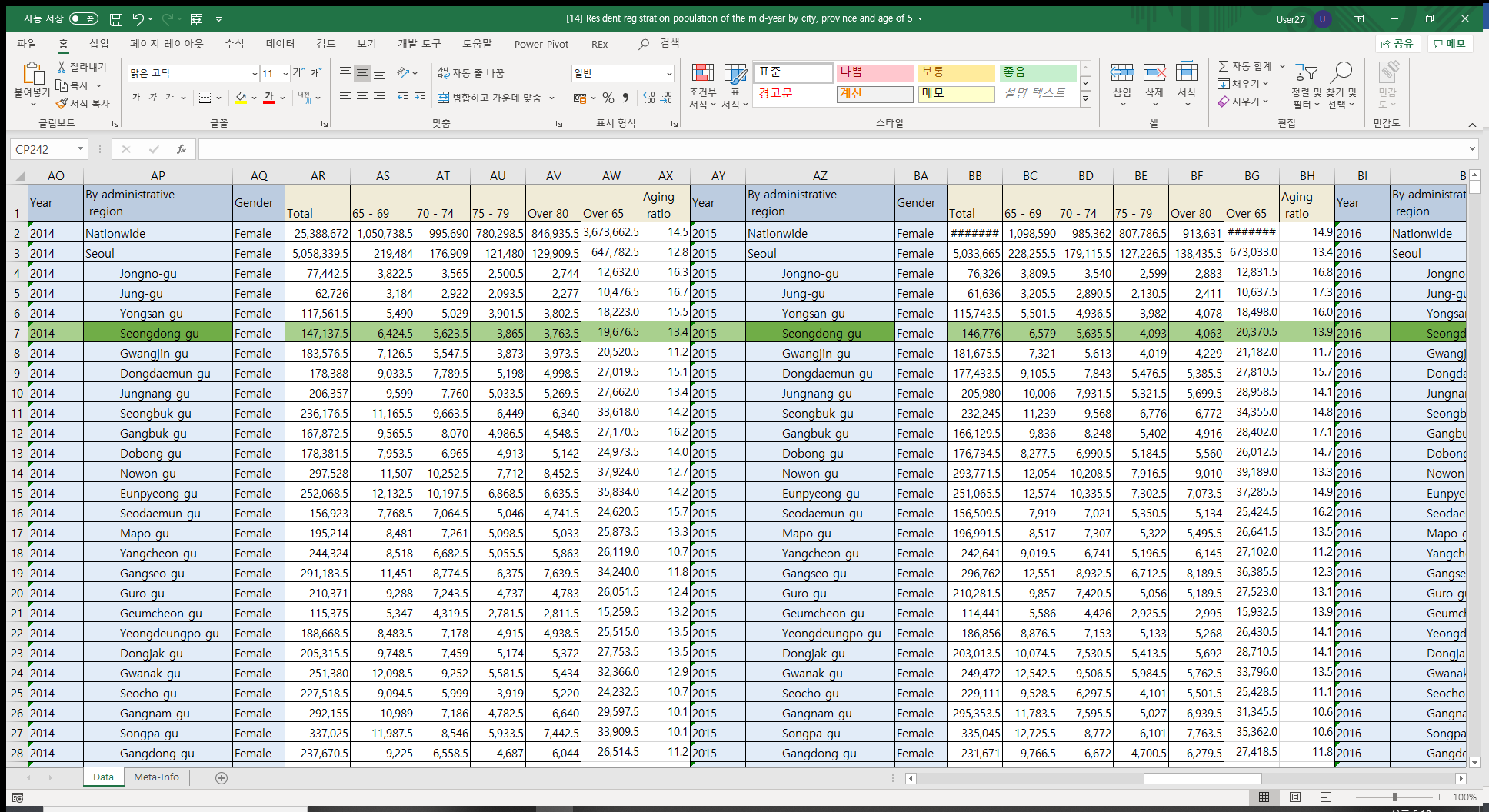


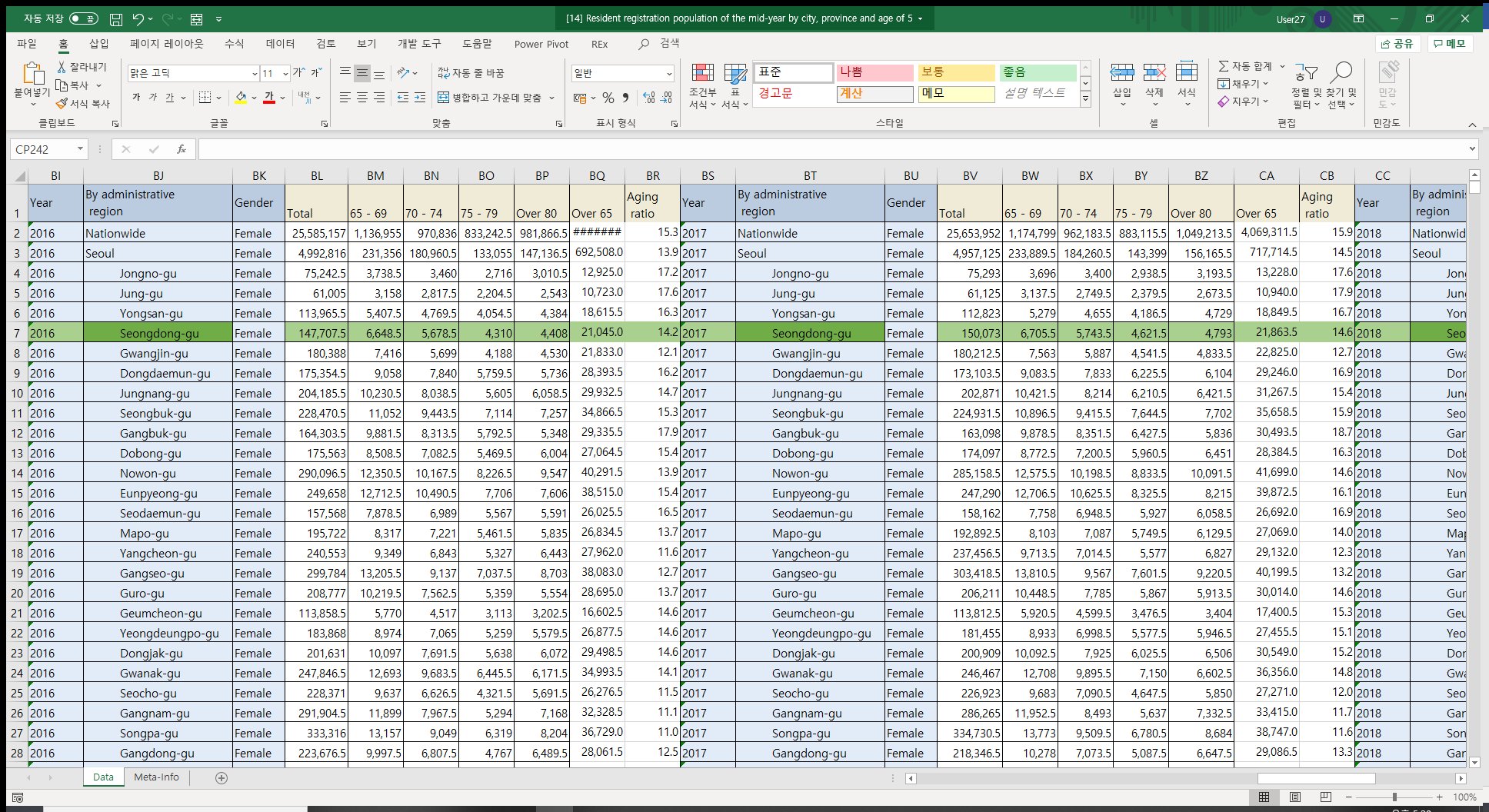


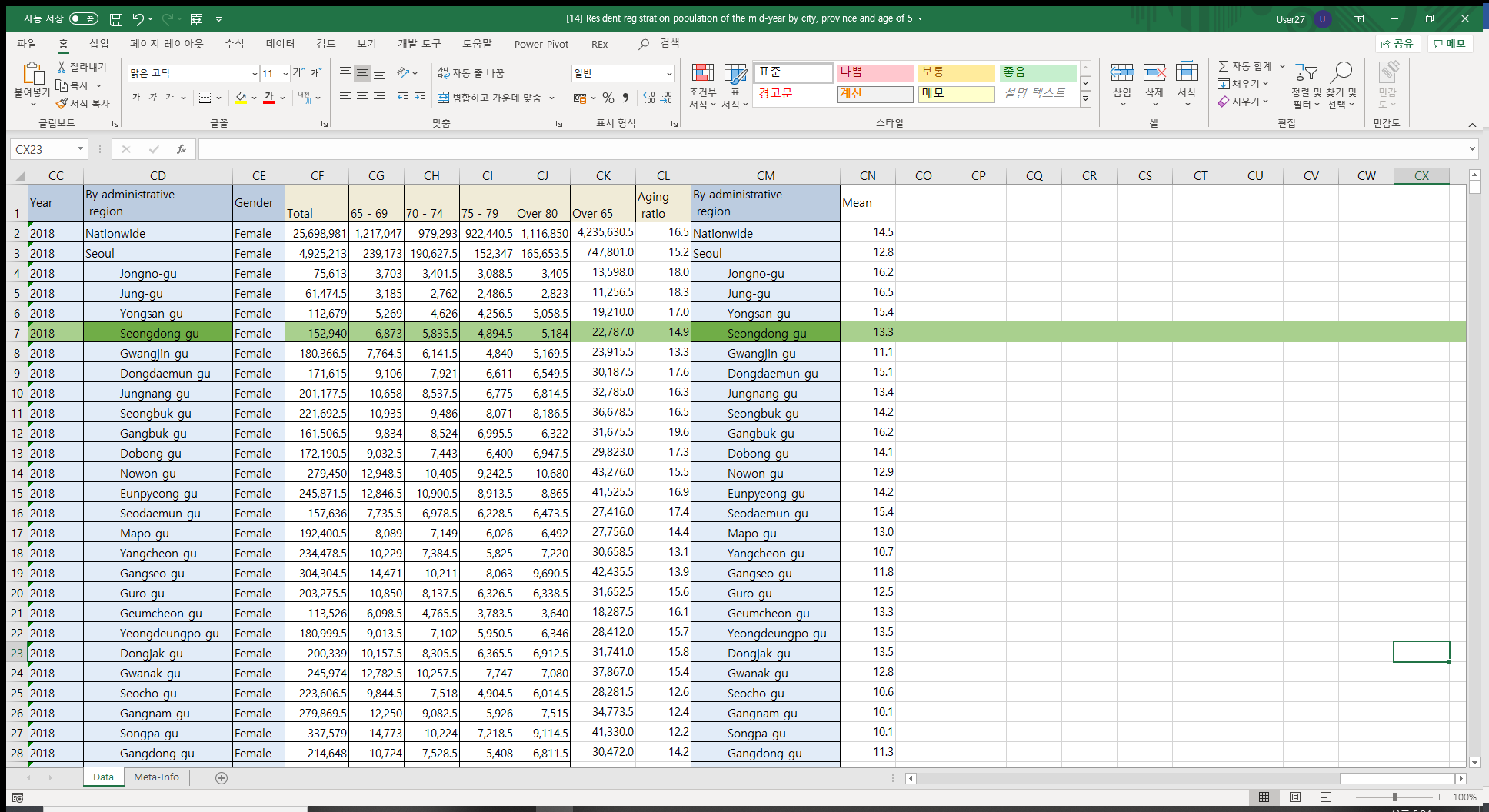


# S4 Dataset

a. original refence: [15] Gender life transition period (66 years) by city, county and district

b. The dataset’s mean data for 2018 is obtained from the average of 2010 ~ 2017.


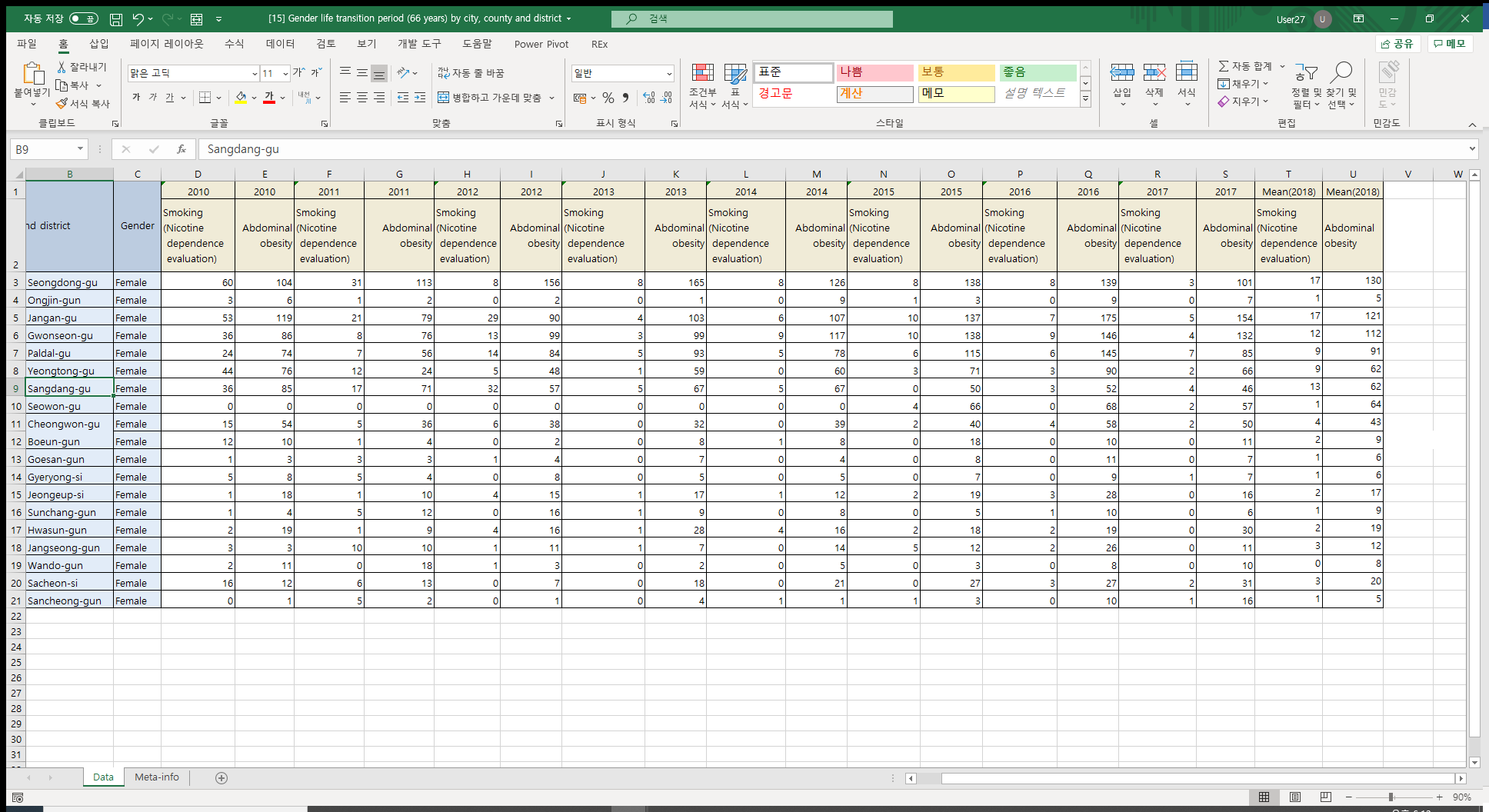


# S5 Dataset

a. original refence: [16] Alcohol rate by province, city, county, and district


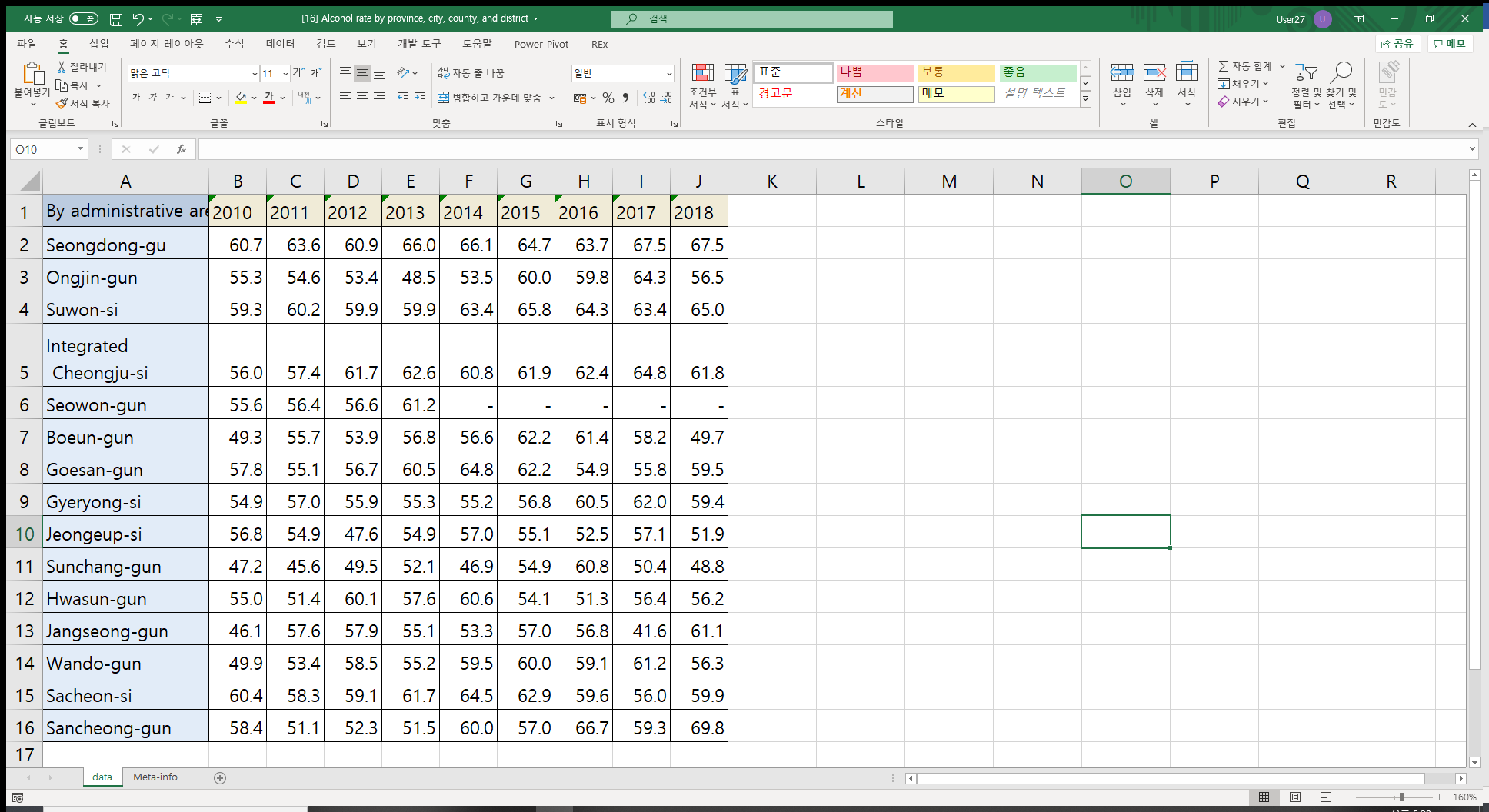


# S6 Dataset

a. original refence: [17] Number of incidences, relative frequency, incidence rate, and age standardized incidence ratio by 24 carcinomas, gender


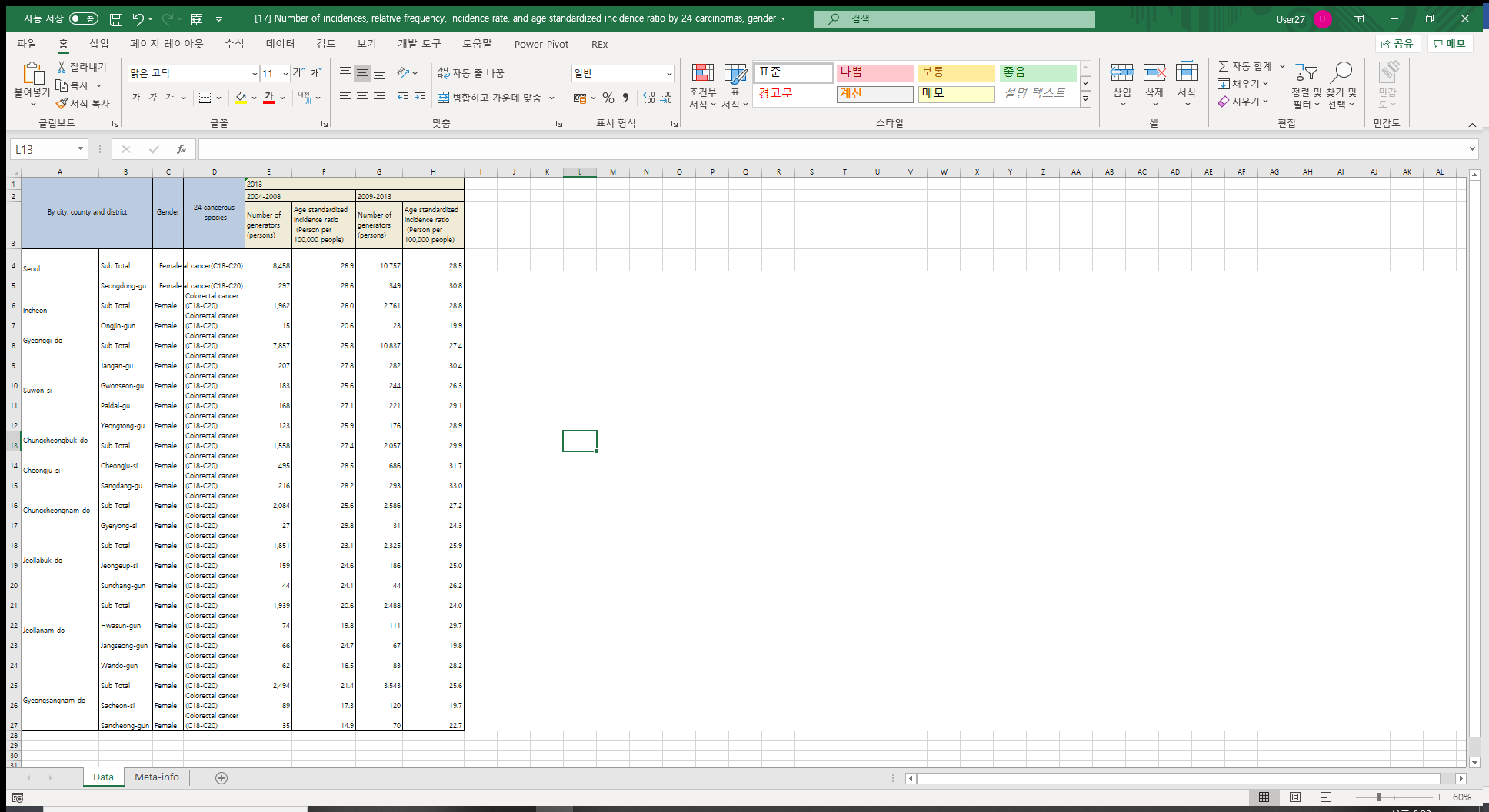


# S7 Dataset

a. refence: [18~24] Total value added and factor income in the region by economic activity

a-1. S7 Dataset is equivalent to S7A to S7G combined. In this paper, only the data of ‘intermediate consumption’ in S11 was used.


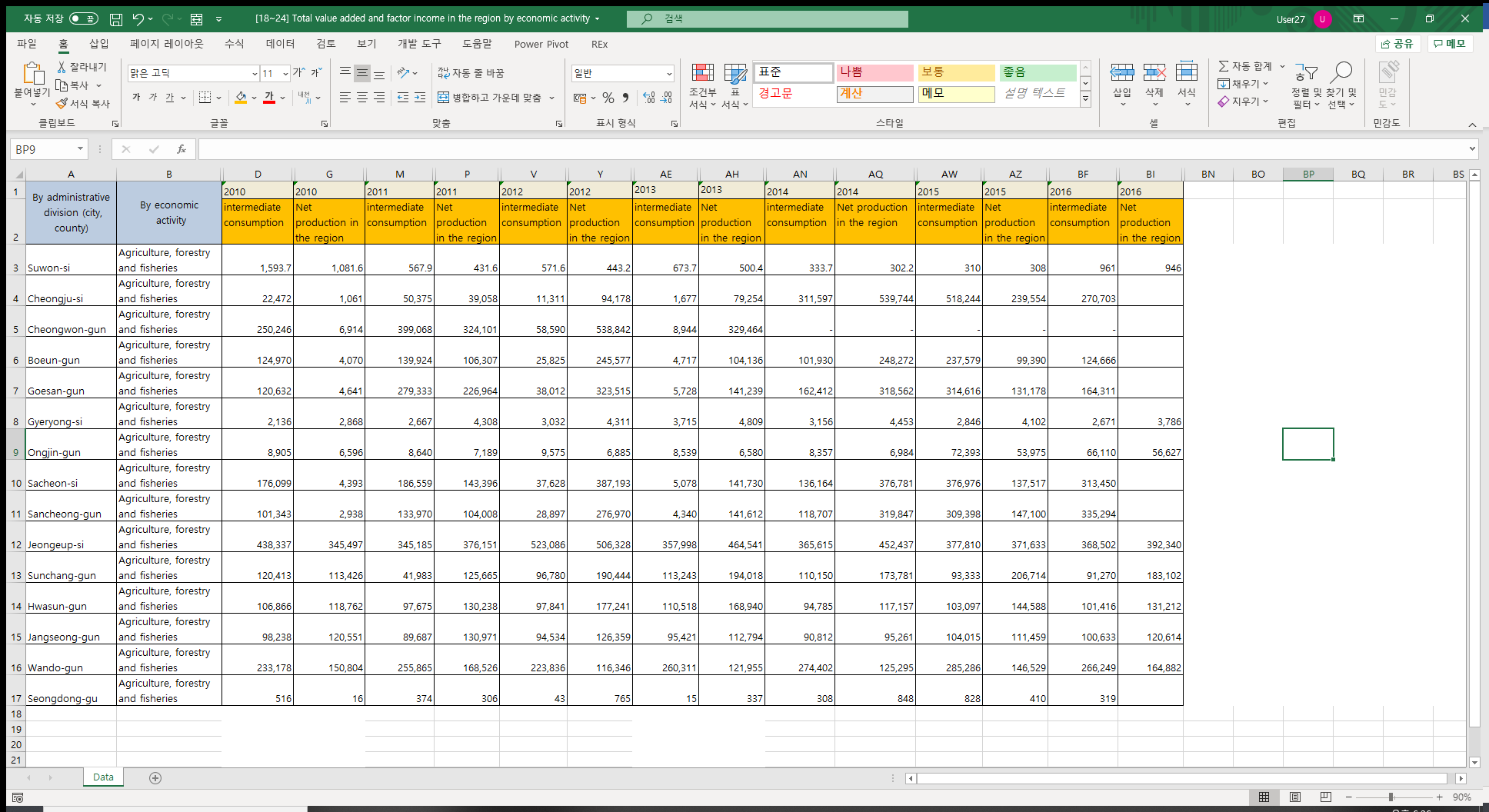


S7A Dataset

b-1. original refence: [18] Total value added and factor income in the region by economic activity in Gyeonggi-do


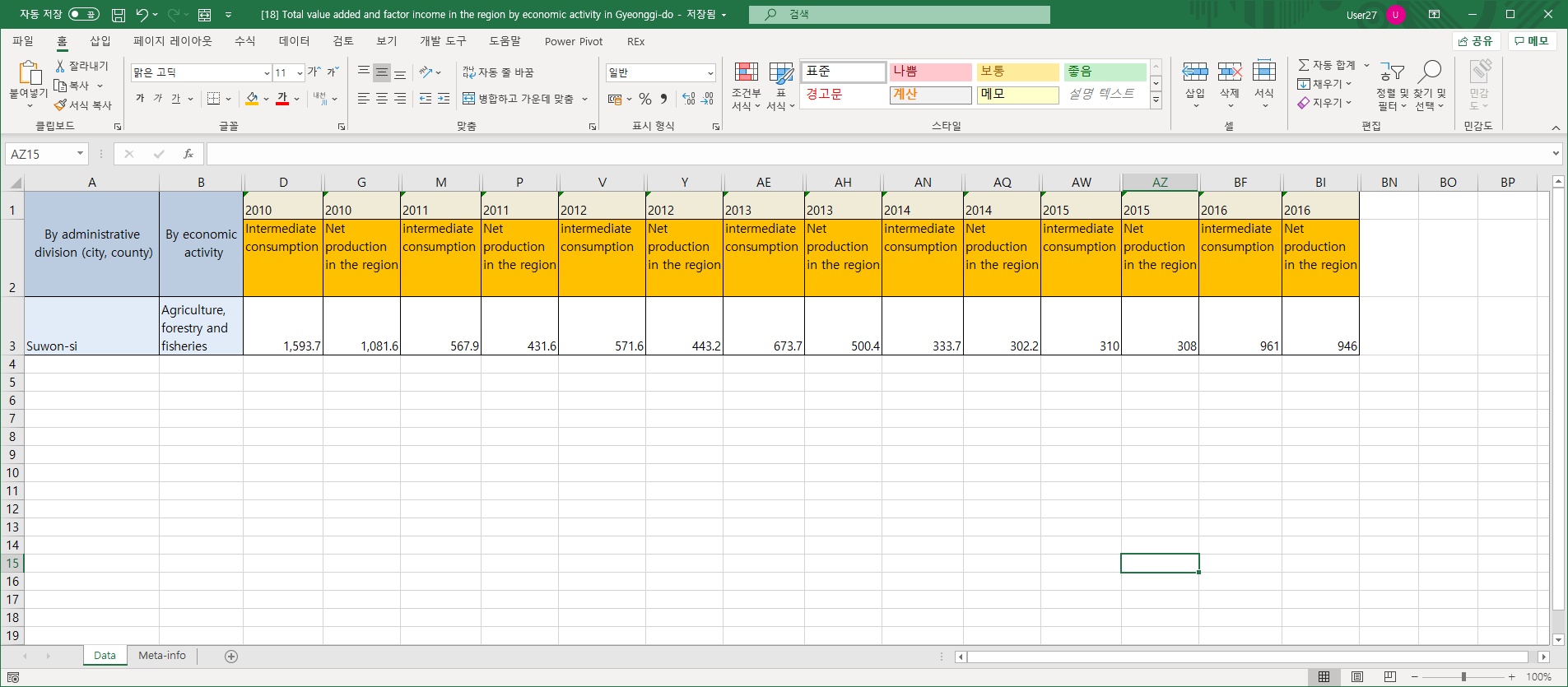


## S7B Dataset

c-1. original refence: [19] Total value added and factor income in the region by economic activity in Gyeongsangnam-do


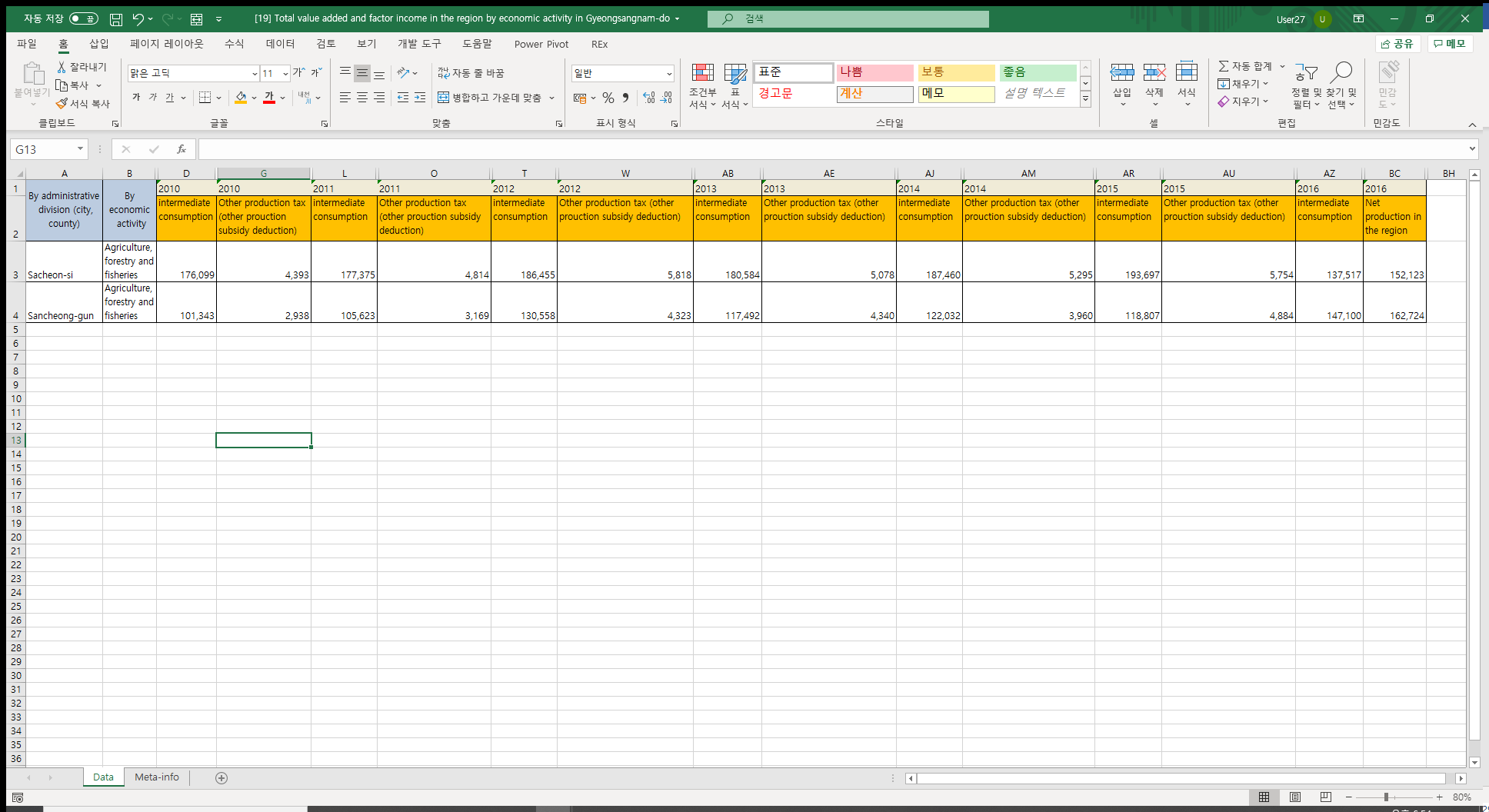


## S7C Dataset

d-1. original refence: [20] Total value added and factor income in the region by economic activity in Seoul


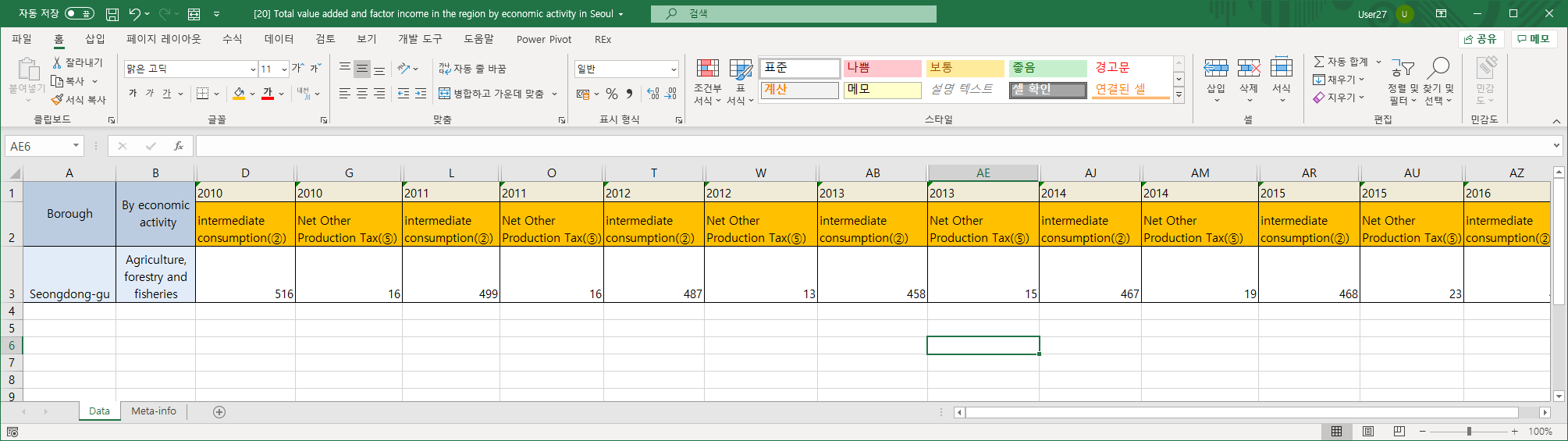


## **S7D Dataset**

e-1. original refence: [21] Total value added and factor income in the region by economic activity in Incheon metropolitan City


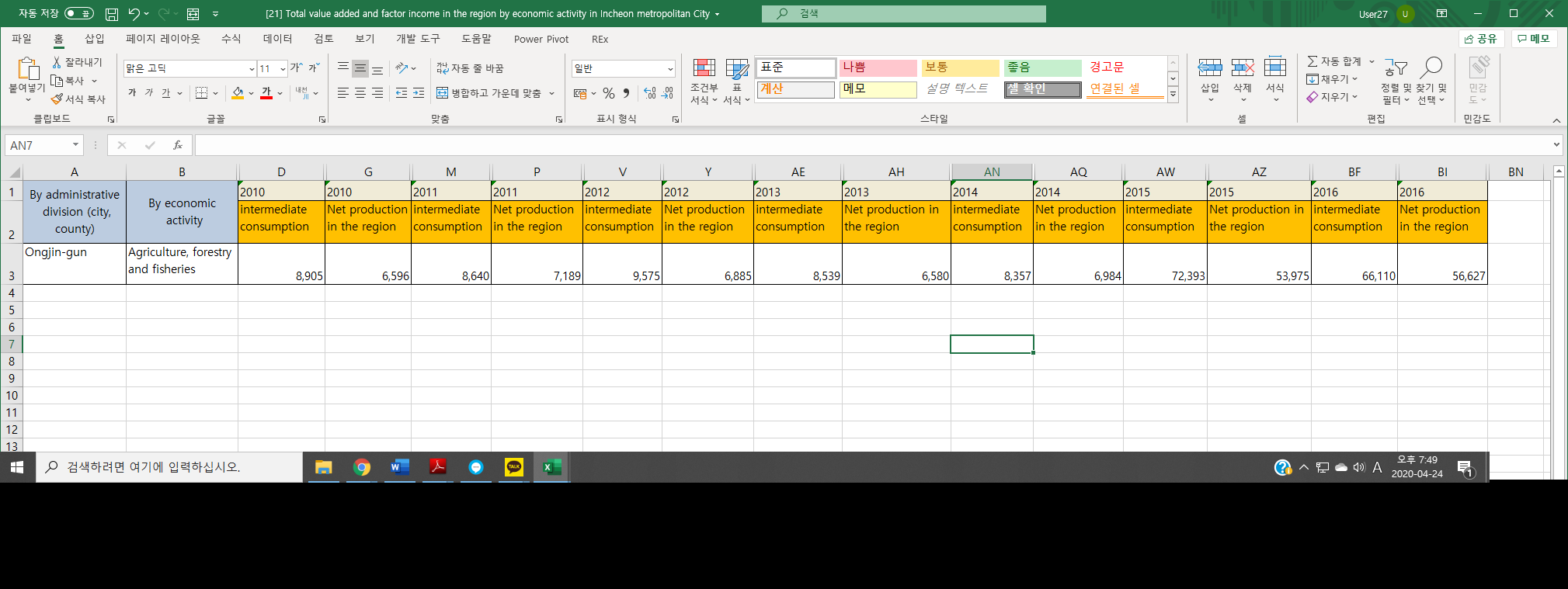


## S7E Dataset

f-1. original refence: [22] Total value added and factor income in the region by economic activity in Jeollanam-do


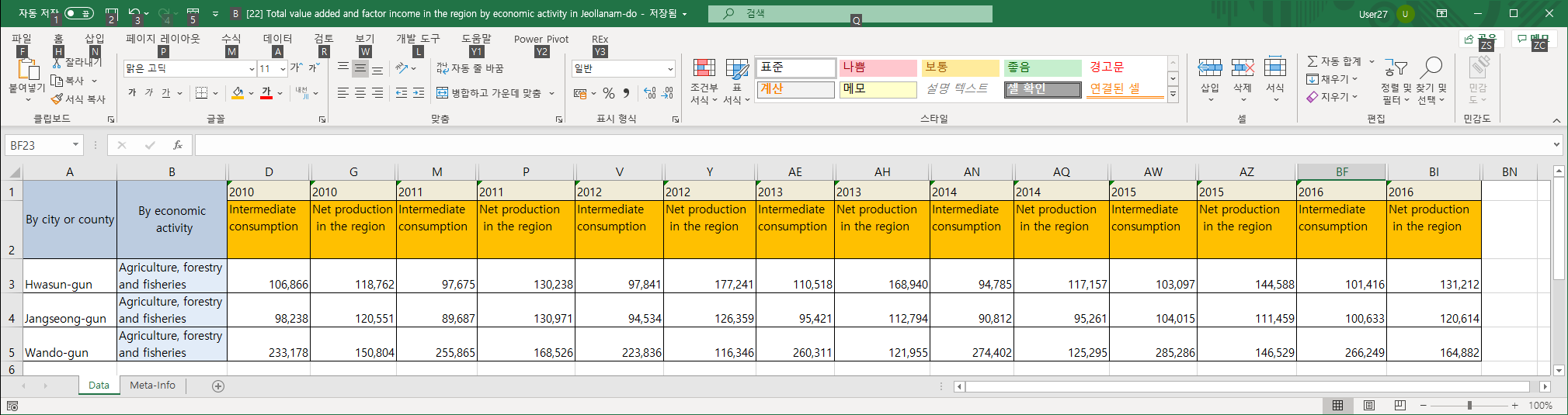


## S7F Dataset

g-1. original refence: [23] Total value added and factor income in the region by economic activity in Jeollabuk-do


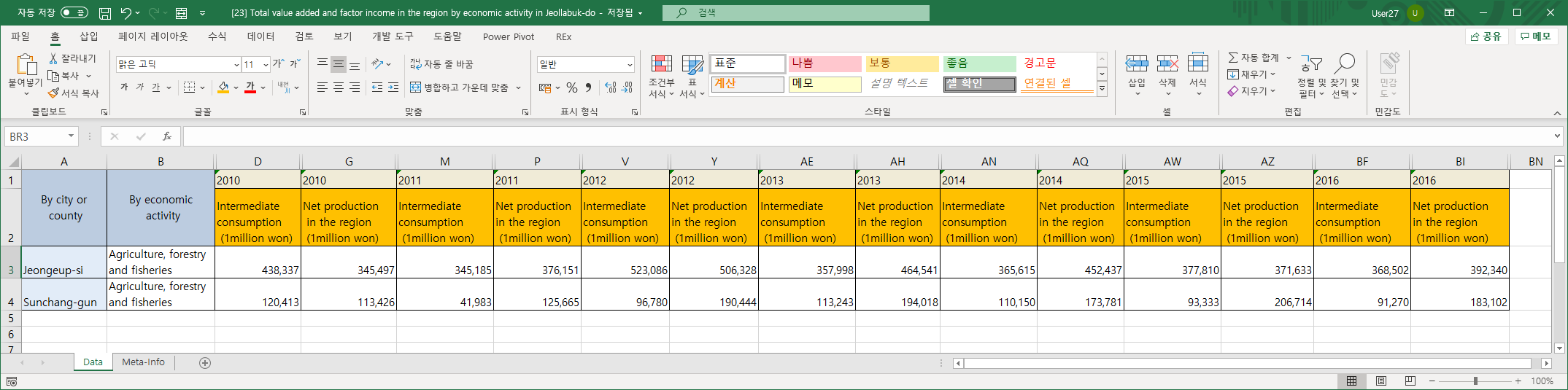


## S7G Dataset

h-1. original refence: [24] Total value added and factor income in the region by economic activity in Chungcheong buk-do


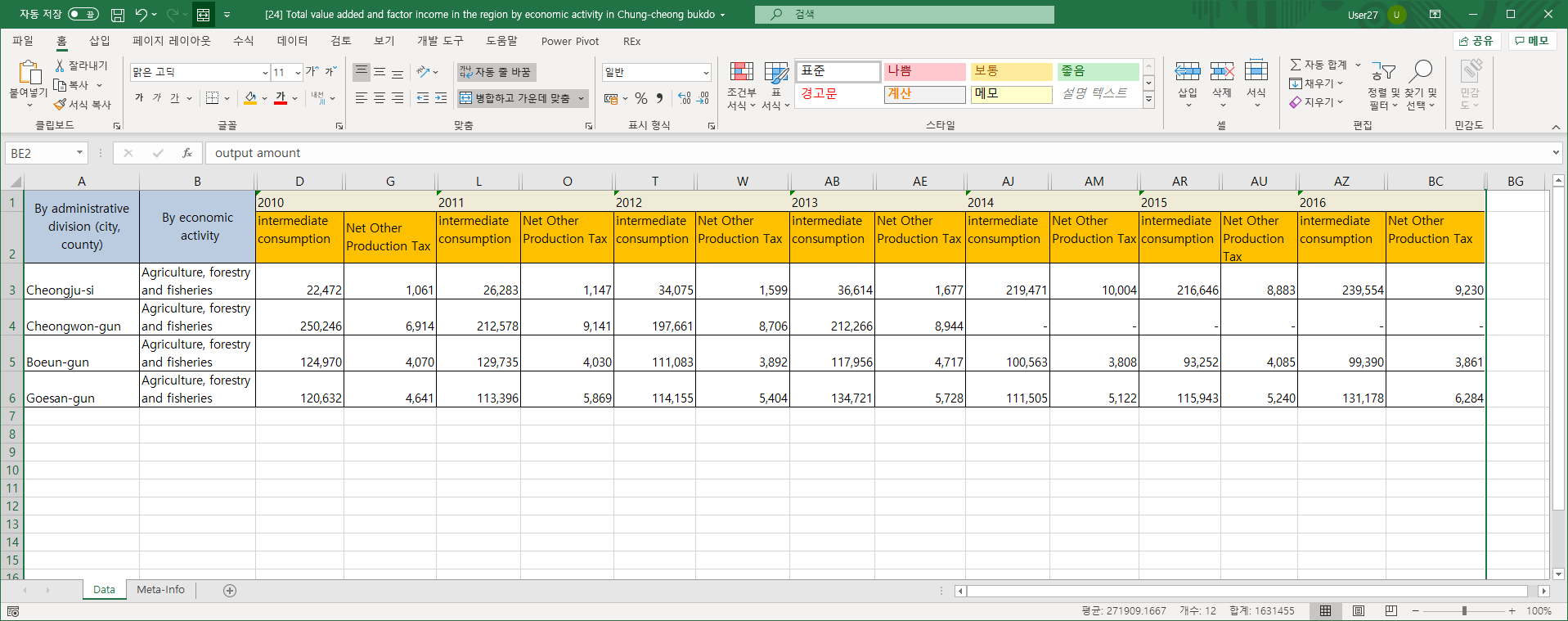


# S8 Dataset

a. original refence: [25] Eco-friendly certified agricultural products shipment status by province, city, country and district


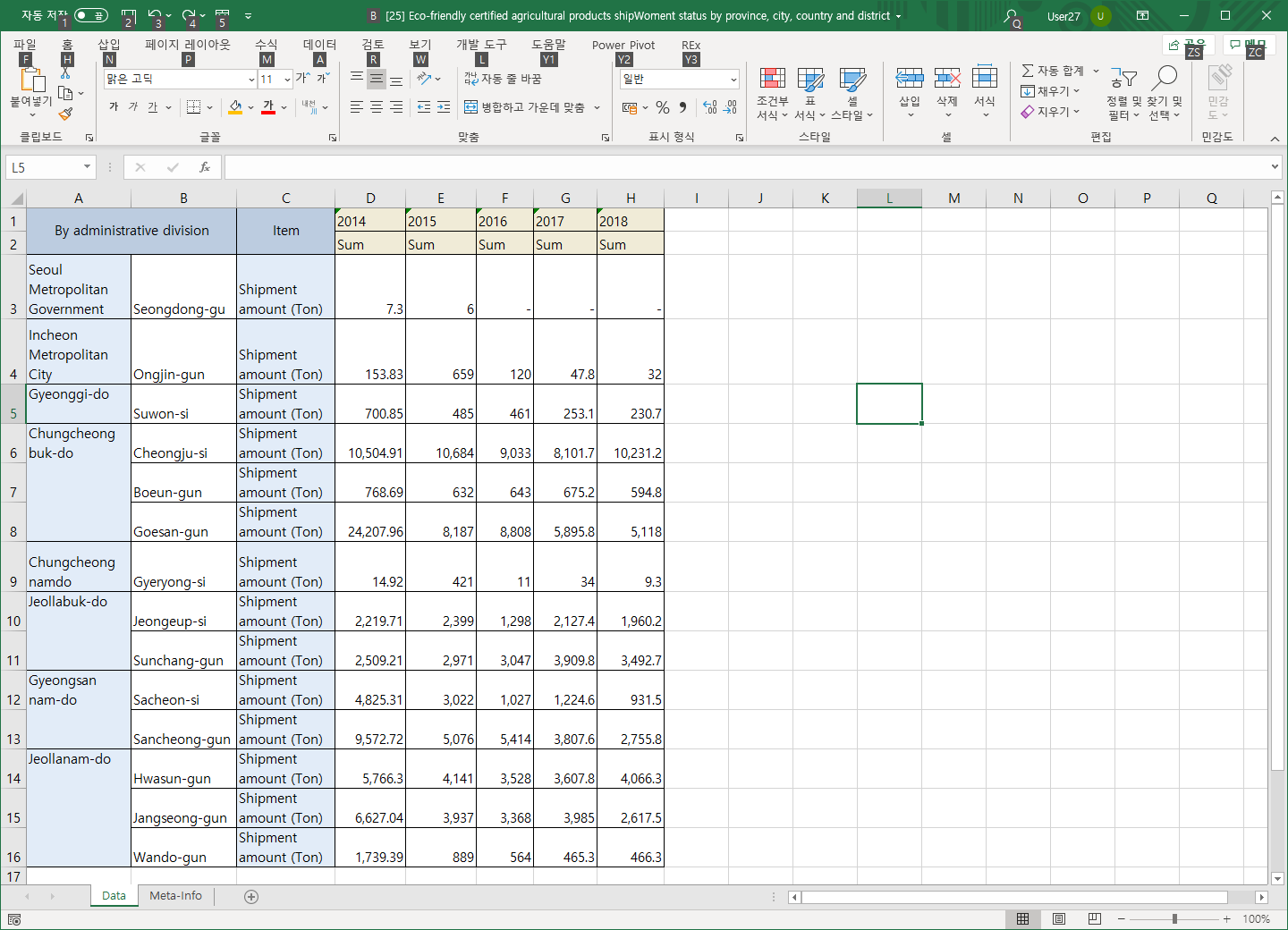


# S9 Dataset

a. original refence: [26] Production volume and amount by product and by province, city, county and district


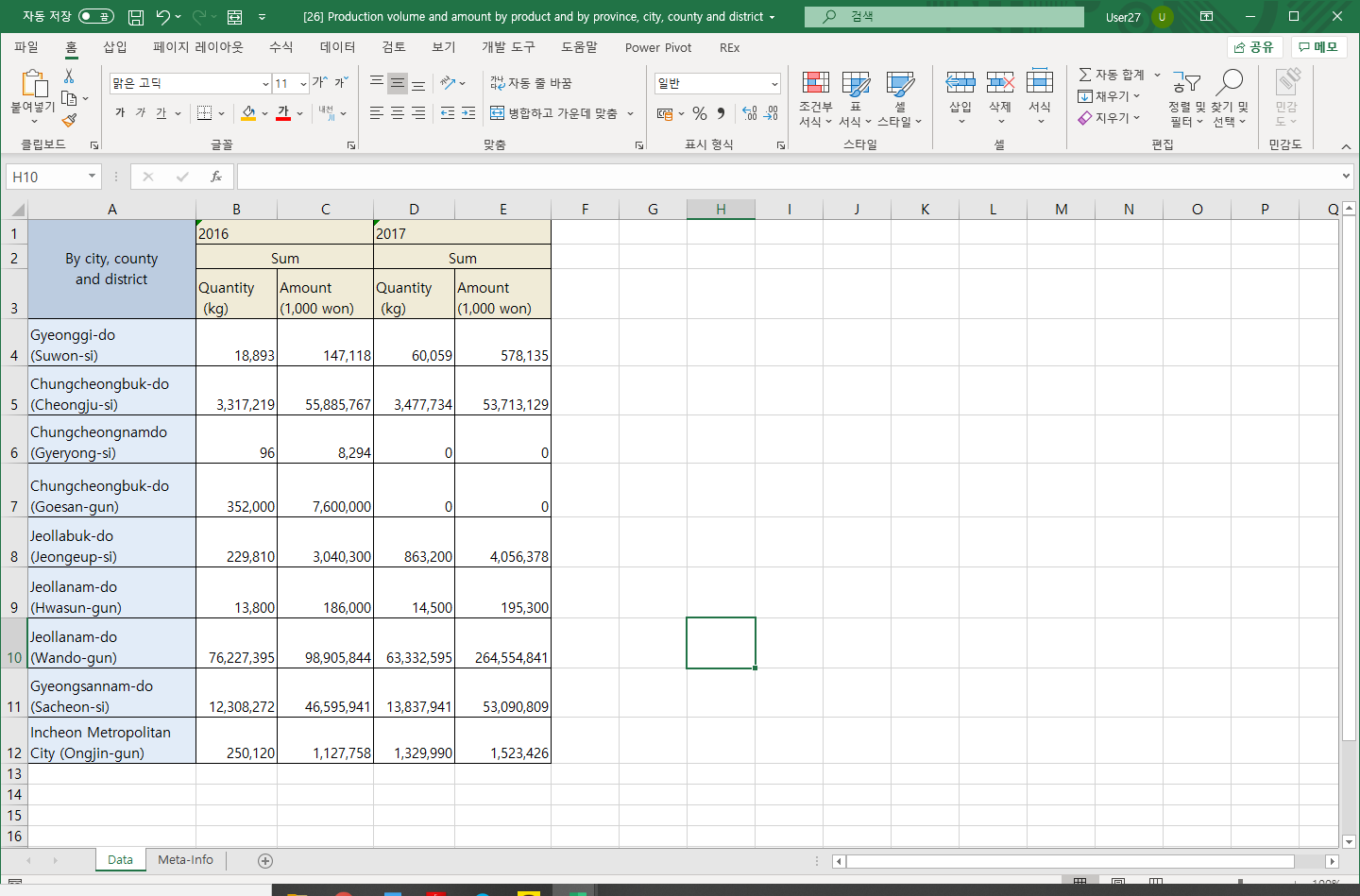


# S10 Dataset

a. original refence: [27] Eco-friendly certified livestock products shipment status by province, city, county and district

b. Among the items in the dataset below, ‘Shipment (Ton)’ was used only in S11 Dataset.


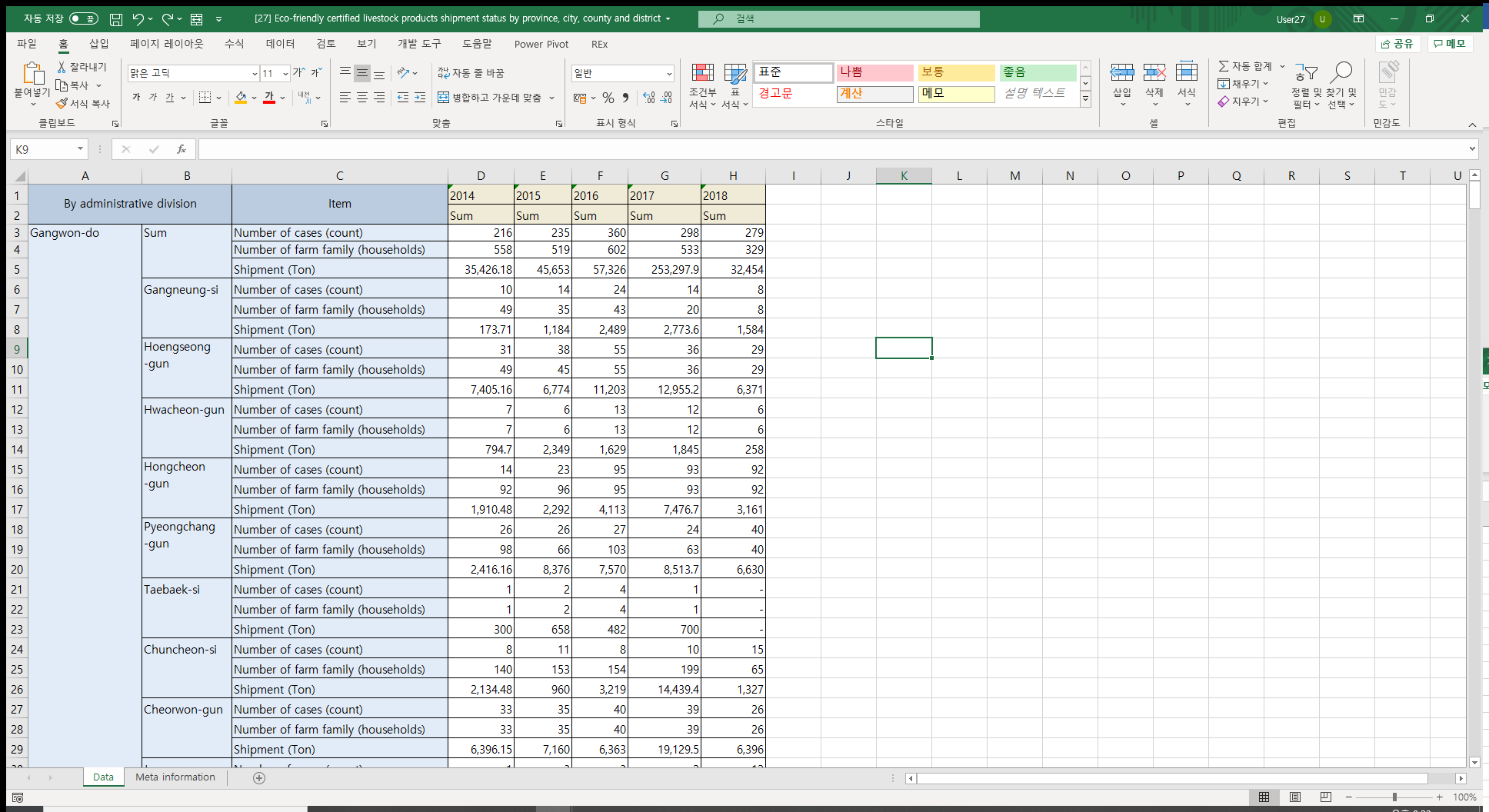


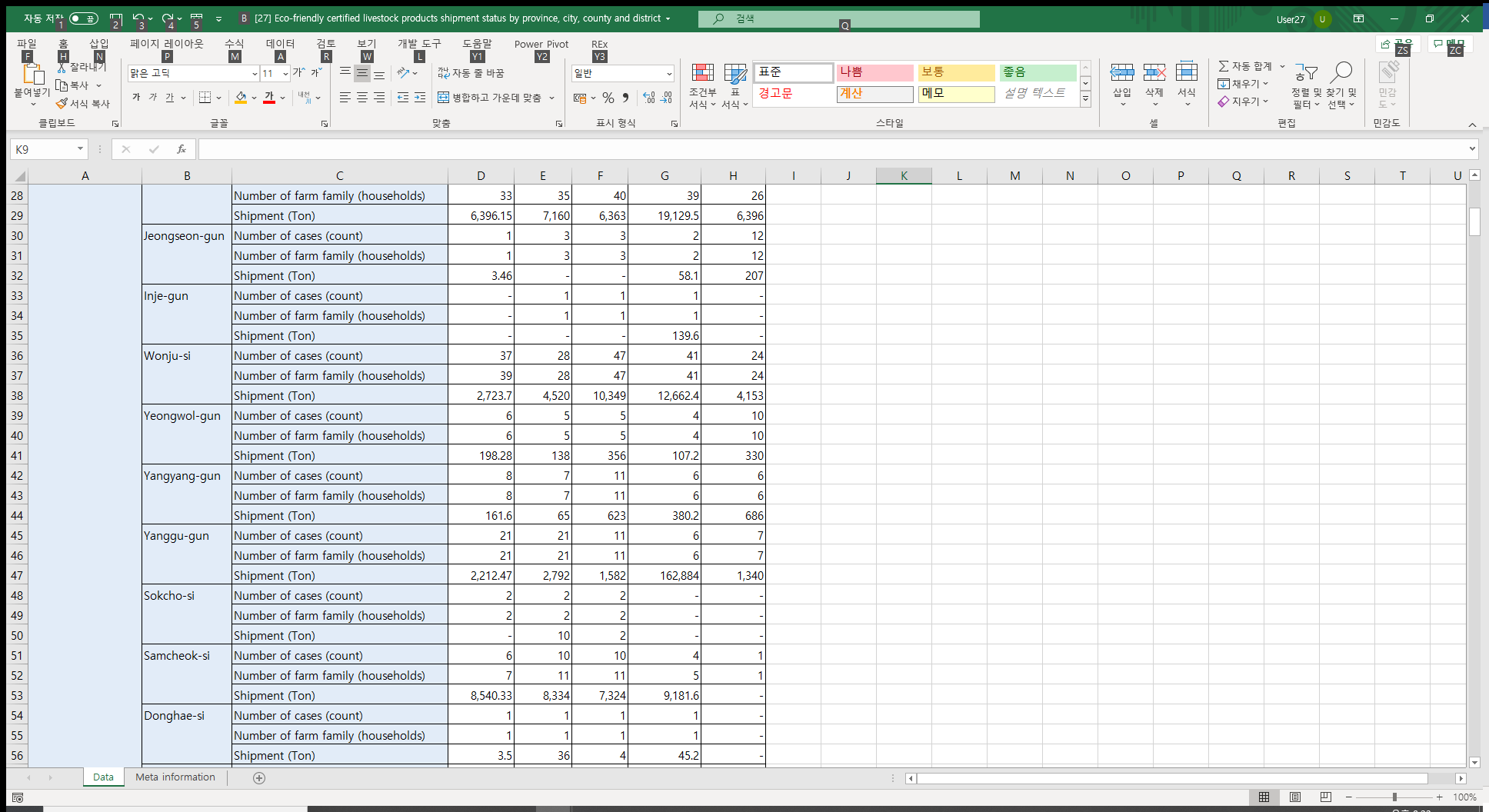


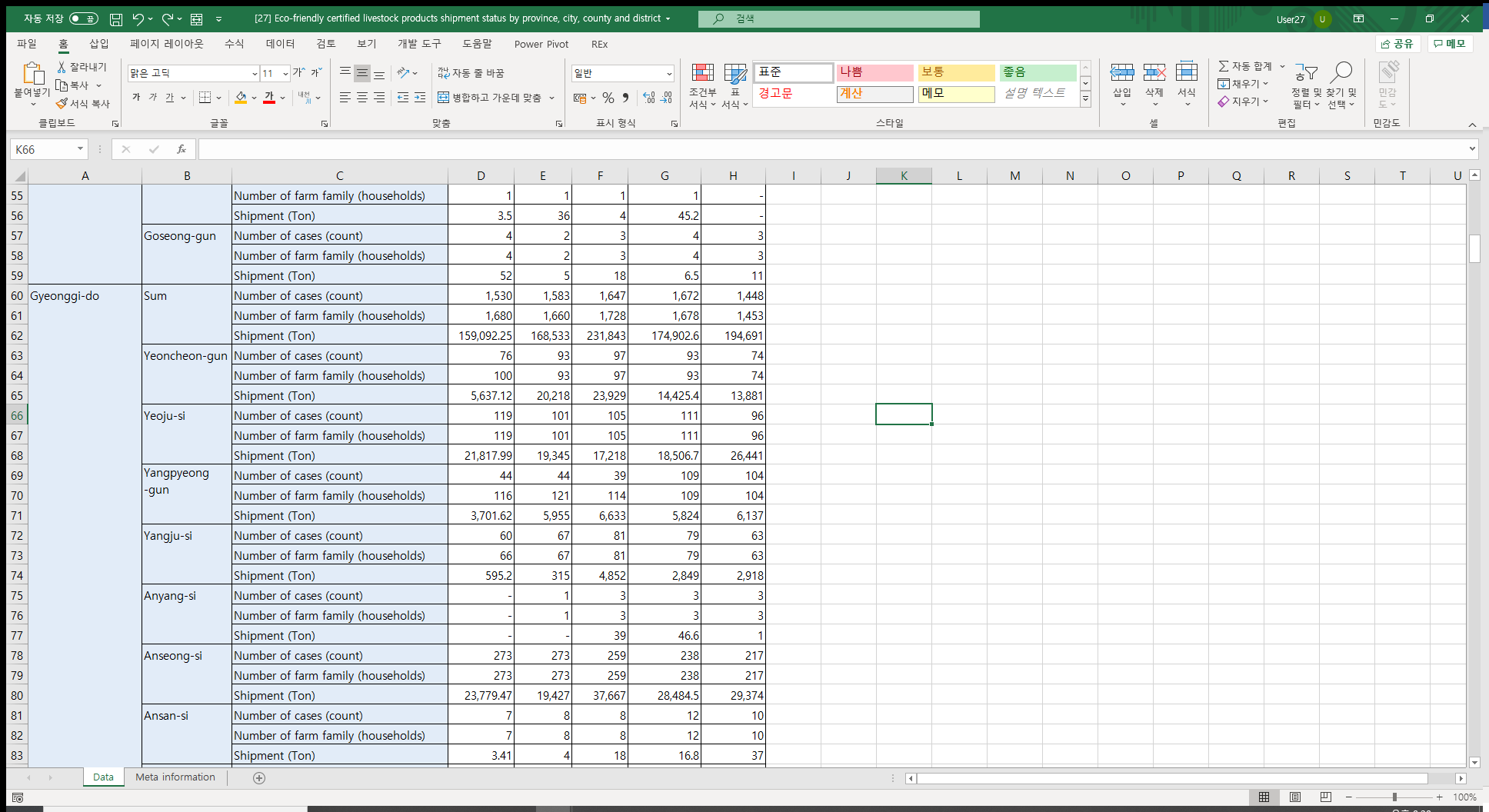


# S11 Dataset

a. Original file name: SiGunDeath_TLSCCO10_2.csv

b. The configuration of this dataset is as follows.

| Column of S11 Dataset | Dataset No. | Reference No | Statistical Table ID in Meta information |
| --- | --- | --- | --- |
| Can | S2D Dataset | [13] | 250 CRC sheet, DT_1B34E13 |
| Total_pop | S3 Dataset | [14] | DT_1B040M5 |
| 65old | S3 Dataset | [14] | DT_1B040M5 |
| Mortality | S2A Dataset | [13] | Data sheet, DT_1B34E13 |
| 66_Abdom | S4 Dataset | [15] | DT_35007_N029 |
| C50_ASR_D | S2B Dataset | [13] | Data sheet, DT_1B34E13 |
| E1014_ASRD | S2C Dataset | [13] | Data sheet, DT_1B34E13 |
| C1820_ASRI | S6 Dataset | [17] | DT_117N_A11109 |
| 65old_Pop | S3 Dataset | [14] | DT_1B040M5 |
| 66_Smoke | S4 Dataset | [15] | DT_35007_N029 |
| AlcoholRate | S5 Dataset | [16] | DT_1YL20991E |
| Agriculture2 | S8 Dataset | [25] | DT_1YL20121 |
| Fishing2 | S9 Dataset | [26] | DT_MLTM_5002733 |
| Livestock2 | S10 Dataset | [27] | DT_1YL20131 |
| Consump | S7 Dataset | [18~24] | [18] DT_GRDP201031_02  [19] DT_GRDP201038_02  [20] DT_GRDP201011_02  [21] DT_GRDP_02_2010  [22] DT_GRDP201036_02  [23] DT_GRDP201035_02  [24] DT_GRDP201033_02 |


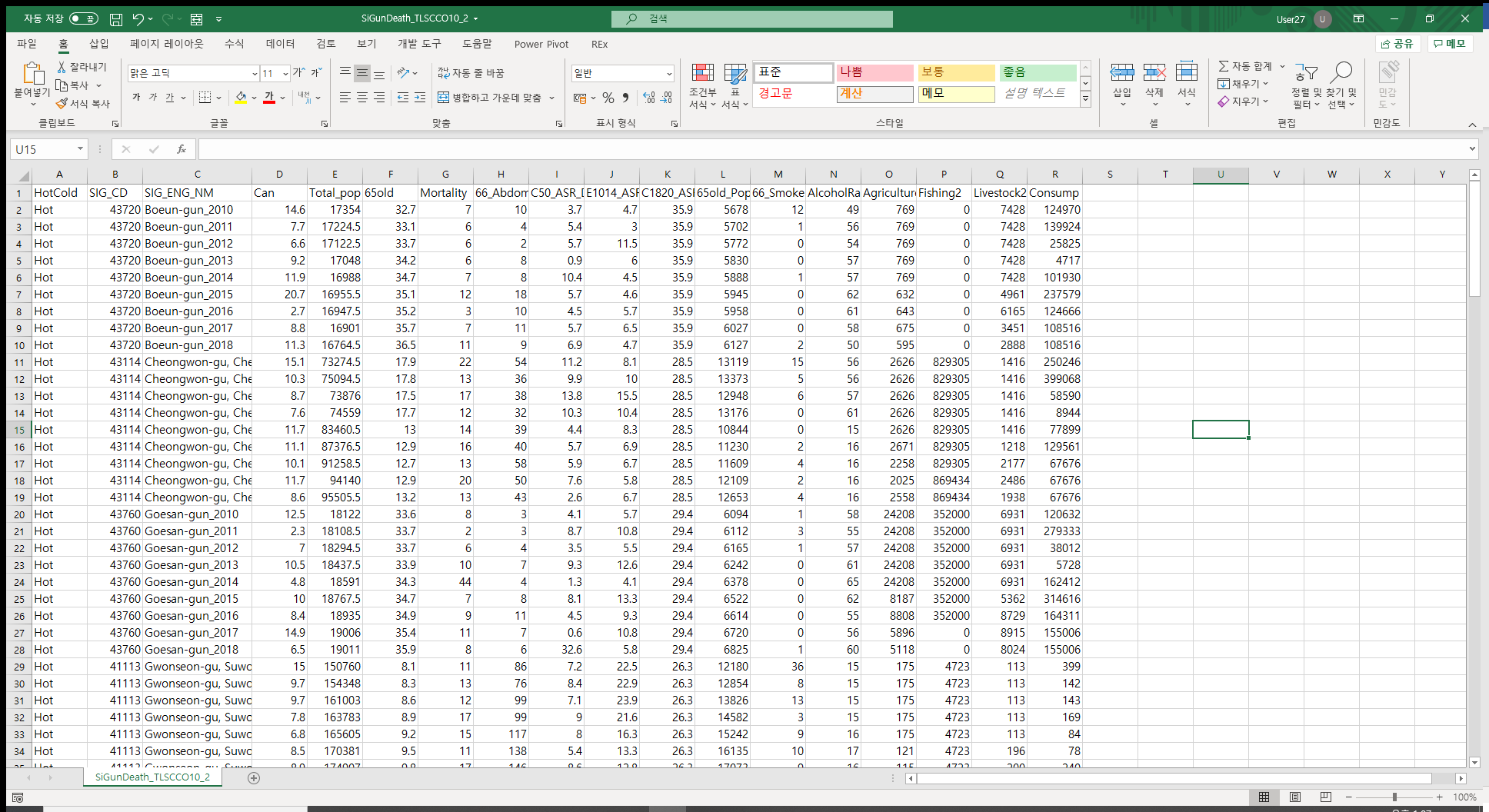


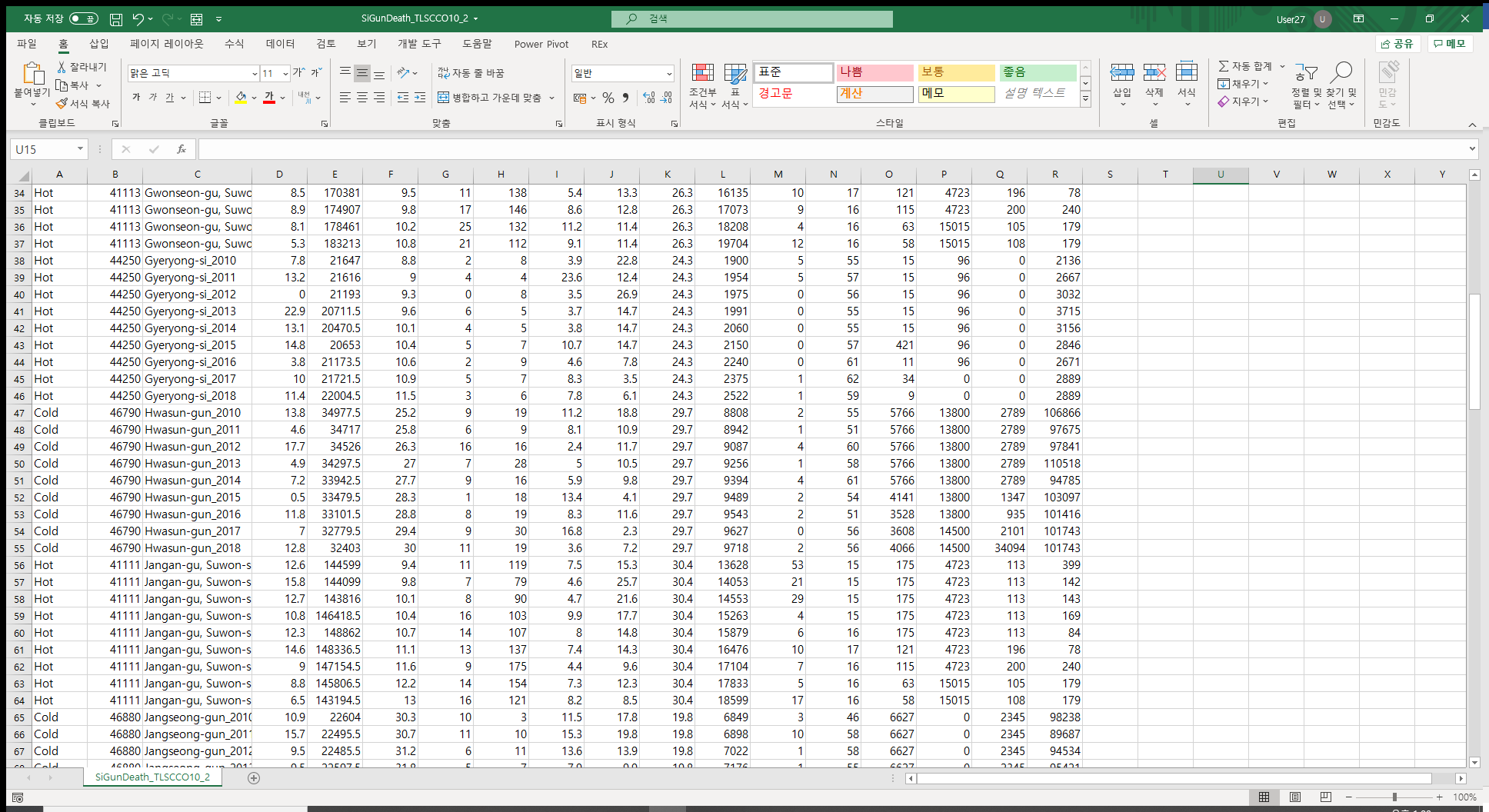


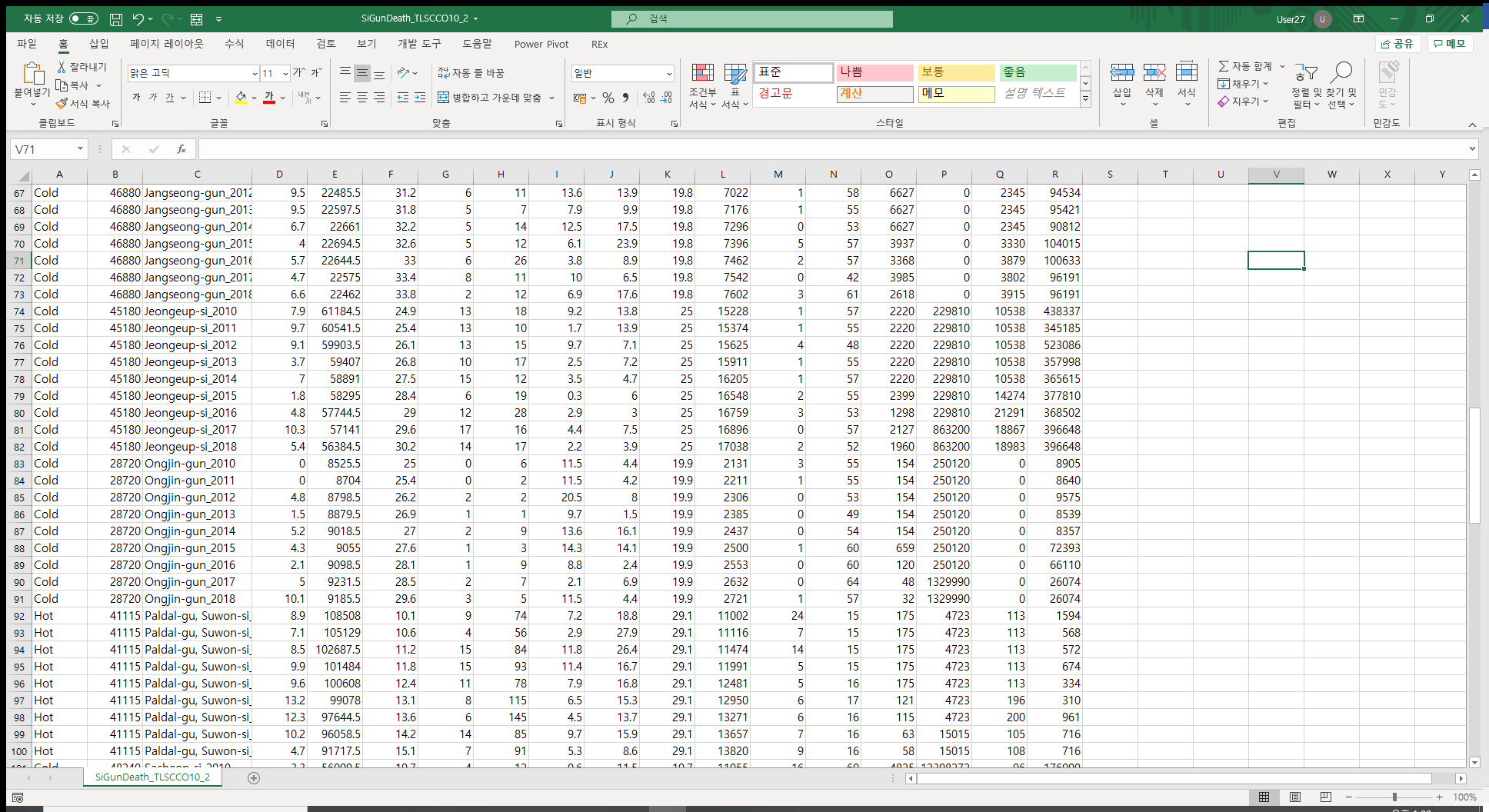


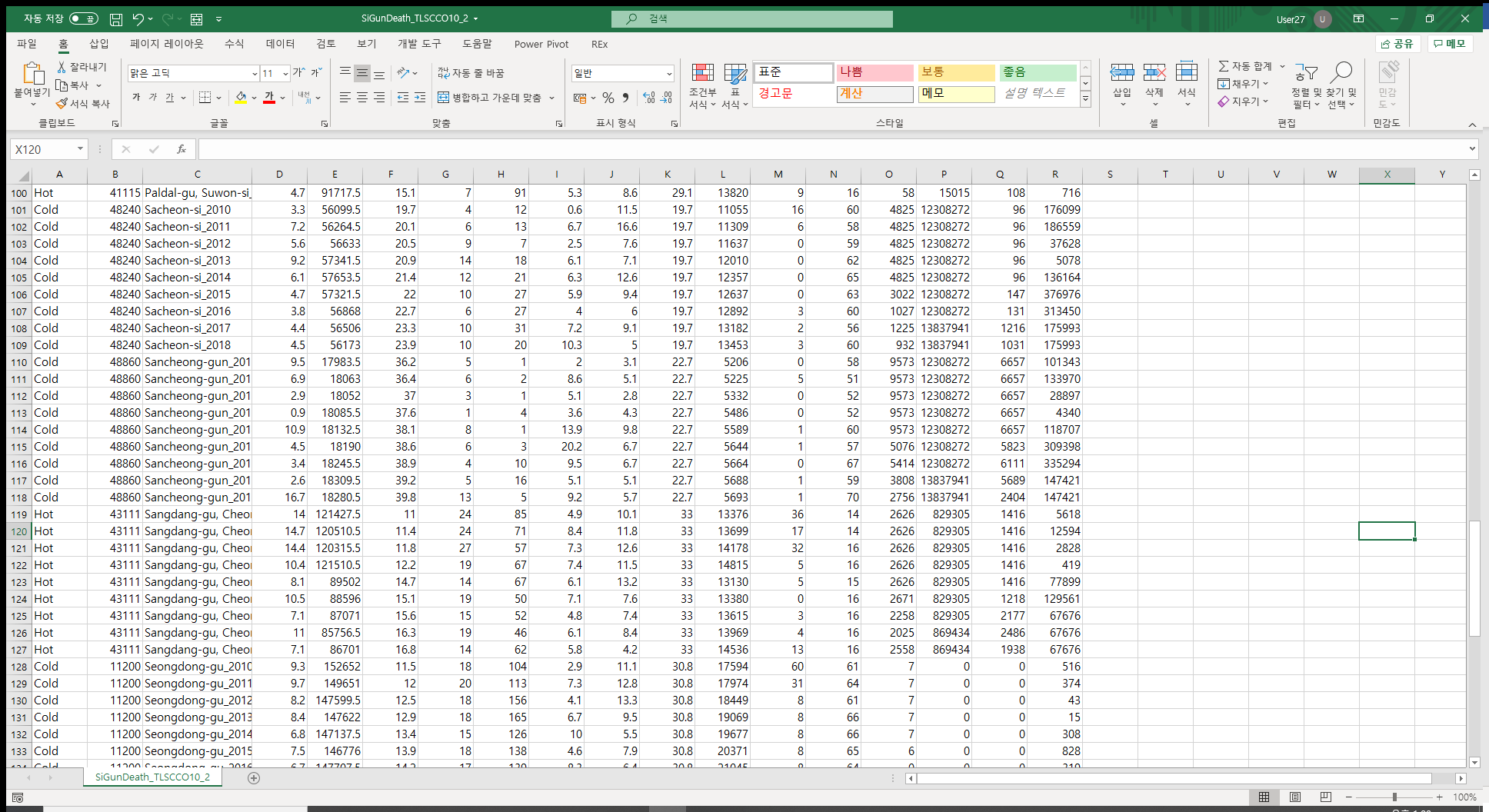


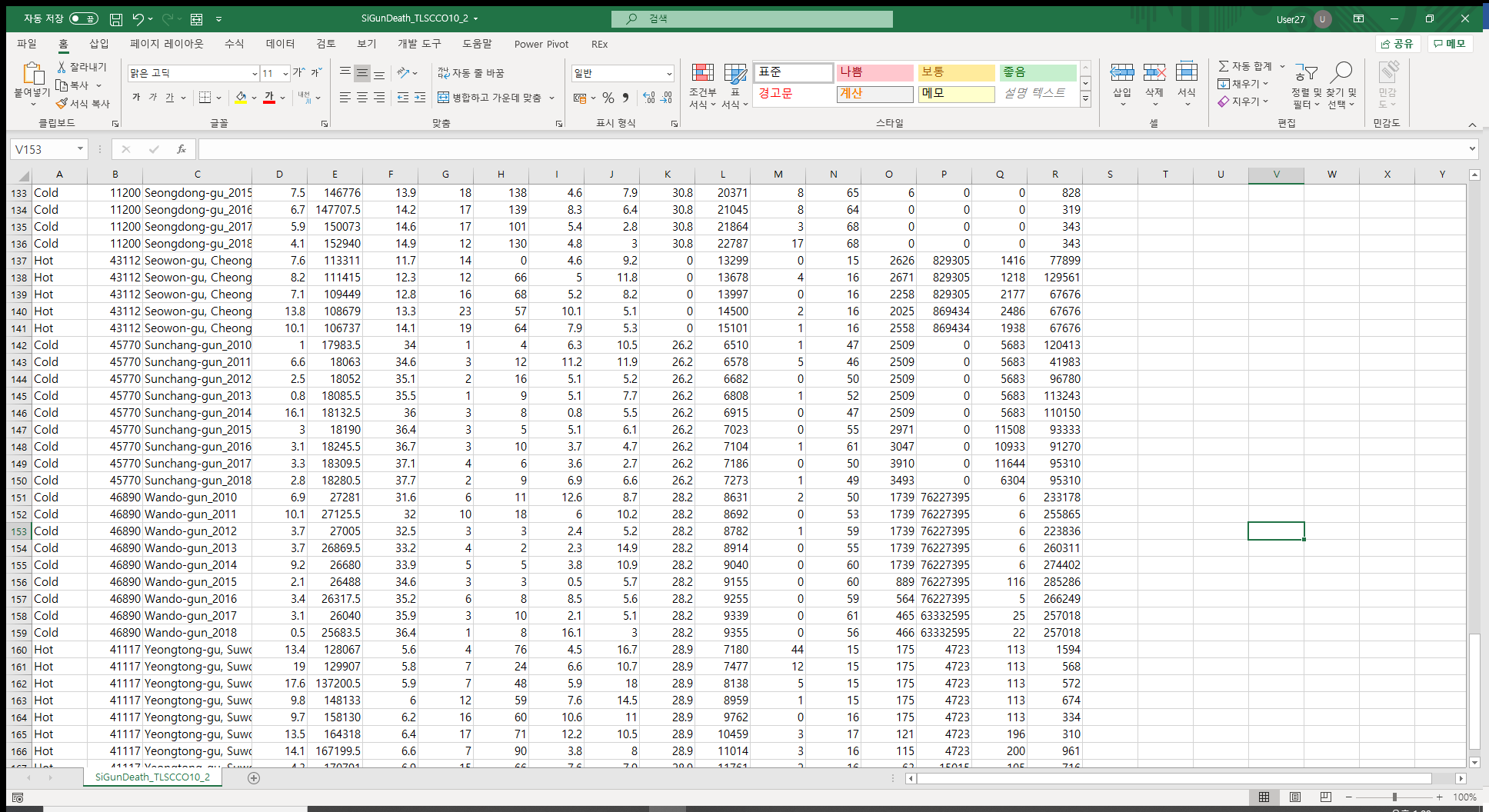


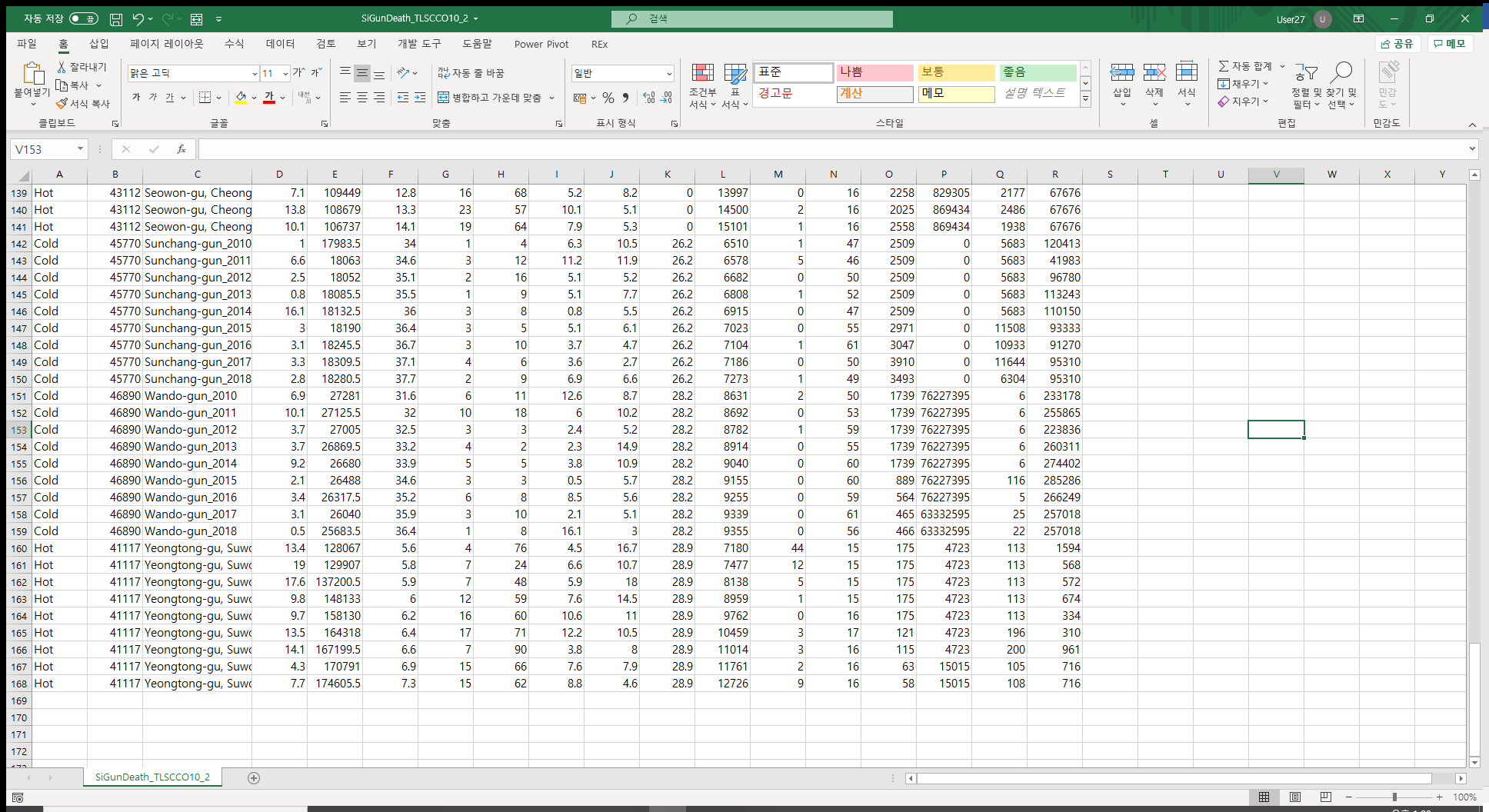

Supplement: S1 File — (DOCX) [file pone.0273995.s001.docx]
